# Exploring geriatric trauma unit experiences through patients’ eyes: a qualitative study

**DOI:** 10.1186/s12877-024-05023-z

**Published:** 2024-05-30

**Authors:** Kristen Molendijk-van Nieuwenhuyzen, Renske Belt-van Opstal, Lysette Hakvoort, Jeroen Dikken

**Affiliations:** 1 Franciscus Gasthuis & Vlietland, Kleiweg 500, Rotterdam, 3004 BA The Netherlands; 2 Máxima MC, de Run 4600, Veldhoven, 5504 DB The Netherlands; 3 De Haagse Hogeschool, Faculteit Gezondheid, Voeding & Sport, Johanna Westerdijkplein 75, 2521 EN The Hague, The Netherlands

**Keywords:** Comanaged Care Model, Geriatric Trauma Unit, Fractures, Orthogeriatrics, Fall-related injury, Patient experience, Patient-Centered Care

## Abstract

**Introduction:**

The surgical management of older patients is complex due to age-related underlying comorbidities and decreased physiological reserves. Comanaged care models, such as the Geriatric Trauma Unit, are proven effective in treating the complex needs of patients with fall-related injuries. While patient-centered care is an important feature of these comanaged care models, there has been minimal research dedicated to investigating the patient experience within Geriatric Trauma Units. Therefore, it remains uncertain whether the Geriatric Trauma Unit’s emphasis on a patient-centered approach truly manifests in these interactions. This study explores how patients with fall-related injuries admitted to a Geriatric Trauma Unit perceive and experience patient-centered care during hospitalization.

**Methods:**

This qualitative generic study was conducted in three teaching hospitals that integrated the principles of comanaged care in trauma care for older patients. Between January 2021 and May 2022, 21 patients were interviewed.

**Results:**

The findings highlight the formidable challenges that older patients encounter during their treatment for fall-related injuries, which often signify a loss of independence and personal autonomy. The findings revealed a gap in the consistent and continuous implementation of patient-centered care, with many healthcare professionals still viewing patients mainly through the lens of their injuries, rather than as individuals with distinct healthcare needs. Although focusing on fracture-specific care and physical rehabilitation aligns with some patient preferences, overlooking broader needs undermines the comprehensive approach to care in the Geriatric Trauma Unit.

**Conclusion:**

Effective patient-centered care in Geriatric Trauma Units requires full adherence to its core elements: patient engagement, strong patient-provider relationships, and a patient-focused environment. This study shows that deviations from these principles can undermine care, emphasizing the need for a holistic approach that extends beyond treating immediate medical conditions.

**Supplementary Information:**

The online version contains supplementary material available at 10.1186/s12877-024-05023-z.

## Background

Falls are a considerable public health concern, causing approximately 684,000 deaths annually and ranking as the second leading cause of accidental death worldwide [1]. The likelihood of falls increases with age, due to a combination of physical, sensory, and cognitive impairments, often exacerbated by environments not fully accommodating the needs of older adults [2]. With the population aged 80 or older expanding rapidly, a corresponding increase in fall-related injuries is expected [1, 3, 4].

Addressing the care needs of older patients with fall-induced injuries is intricately challenging due to the intertwined comorbidities and diminished physiological reserves associated with aging, complicating both surgical interventions and postoperative recovery [5–9]. In response, various treatment models of orthogeriatric care have been developed in the last decade to better serve the complex healthcare requirements of this demographic [10, 11]. Comanaged care, where surgical and geriatric expertise converge within specialized units, has been most effective in enhancing patient outcomes [7, 8, 11–24]. Although comanaged care has been implemented under various names [5, 7–10, 12, 13, 17, 19–37] worldwide, its fundamental aim remains consistent: optimizing patient outcomes and improving quality of life.

Despite the documented success of comanaged care from a clinical perspective, limited research has focused on patient experiences, particularly regarding Patient-Centered Care (PCC) within these settings. PCC is a concept central to the comanaged care model, which emphasizes the participation of patients and their informal caregivers in their care and decision-making processes [9, 12, 31]. PCC focuses on individual health needs and stands for care that respects patient experiences, values and choices [38–40].

This study aims to fill this gap by capturing the experiences of older patients with fall-related injuries who have been admitted to a GTU -a comanaged care model. Of particular interest is understanding how patients perceive and experience PCC throughout their hospital stay. By understanding patient perceptions and experiences, the study aims to uncover meaningful insights that can potentially inform and guide improvements in care practices. These insights are anticipated to enhance the alignment of healthcare delivery with goals and desired rehabilitation outcomes of patients, ultimately enriching patient journey within healthcare systems.

## Methods

### Study design

To capture experiences, a generic descriptive study was designed [41]. To facilitate the reporting of results, the consolidated criteria for reporting qualitative studies (COREQ) were used (see additional file 2).

### Participant identification

The study was conducted in the GTUs of three teaching hospitals across the Netherlands.

Older patients with fall-related injuries who were admitted to a GTU and had undergone treatment were asked to participate in the study. Patients were considered eligible to participate if they were ready for discharge, capable of discussing their experiences in Dutch, and able to give informed consent as assessed by a geriatrician, even if they were mildly cognitively impaired. There is extensive evidence to suggest that mildly cognitively impaired patients who can consent to treatment are able to reflect on their experiences [42–44]. Patients with delirium or unable to consent were excluded.

Recruitment began three days postadmission, with patients who met the criteria being informed about the study by the researchers (KMN or RBO), the GTU’s activity counsellor, or a research/senior nurse. Those expressing an interest were further contacted by KMN or RBO for detailed study information and an interview arrangement. When the researchers were uncertain about the participant’s ability to comprehend the informed consent process, the patient was excluded from the study. In total, 31 patients were approached for participation. If they declined, the reasons were recorded (Fig. 1).

**Fig.**
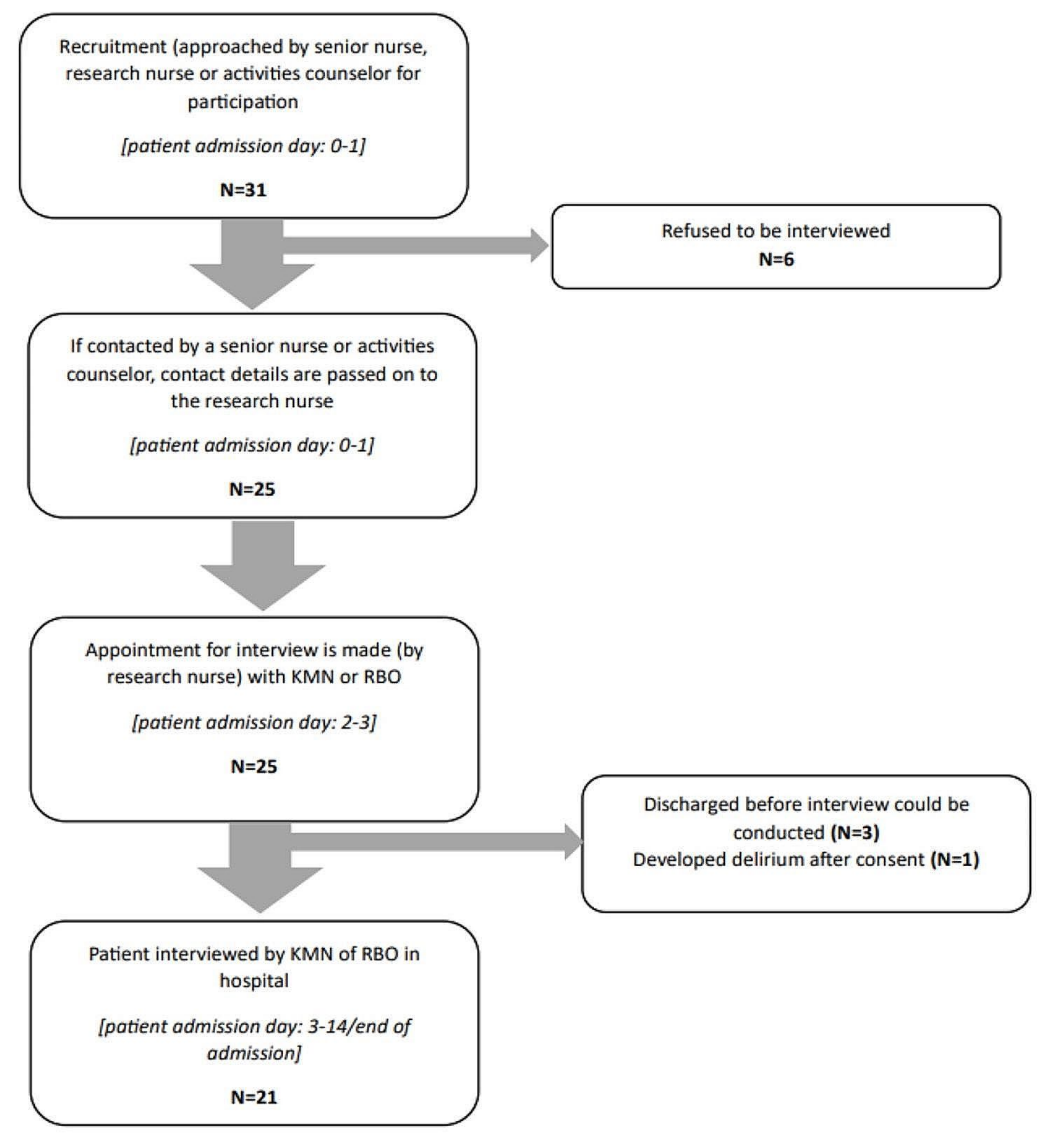
Flowchart patient recruitment

The process of data analysis started in the recruitment phase and continued until data saturation was achieved. Data saturation was deemed attained when subsequent interviews no longer revealed new information pertaining to the research question. The final three interviews yielded no new insights.

### Study procedures

In total, 21 semistructured, in-person interviews were conducted by KMN or RBO in a room that held enough privacy to conduct an interview; all interviewees provided written informed consent before the interview started. Because the researchers were unfamiliar to the participants, they were able to talk freely about their experiences and opinions [45]. Special consideration was given to potential challenges associated with interviewing older patients and appropriate accommodations were made [45–47]. All interviews started with one of two introductory questions: *‘Can you tell me how you came to fall?* Or *‘How are you experiencing the care in a geriatric trauma unit?*’. To ensure the accuracy and confirmability of the participants’ perspectives, interviews ended with a member check by summarizing the content of the interview, soliciting the participant’s agreement and inviting additional contributions. Subsequently, participants were offered the opportunity to receive a transcript of their interview to verify and assess the trustworthiness of the results [45]. However, to lessen the burden of participation, returning transcripts was not standard procedure [46, 48].

The interviews followed a semi structured guide rooted in Gerteis et al.’s [49] eight dimensions of PCC. The interview guide, initially designed by KMN and reviewed by the research team, was refined after 12 interviews to better suit the patient demographics, ensuring thoroughness [45].

Each interview was recorded, with accompanying field notes for observations and data analysis. Detailed memos were produced for preliminary coding. Interview durations ranged from 24 to 70 min.

### Data analysis

Data analysis was completed using a-priori thematic analysis as described by Braun & Clarke [50, 51]. After each interview, field notes and audio recordings were transcribed verbatim, checked, pseudonymized and uploaded into ATLAS.ti, a qualitative software program. After four interviews, the entire research team independently familiarized themselves with the data. This preliminary coding phase involved both descriptive and latent coding techniques, with subsequent data comparison.

While the frame of reference is Gerteis’ PCC framework, as it formed the topic list, the codes were data driven. The consecutive transcripts were independently double-coded by KMN and RBO to strengthen the study and provide insight into the coding process [45, 51].

The codebook was drafted iteratively during data collection and discussed several times with the research team. Braun & Clarke’s checklist was used as a guideline [51]. Throughout the process of data collection and analysis, notes were taken, and decisions were recorded, creating an audit trail [45]. Quotations were selected to bring the reader into a closer understanding of the patient’s perspective [51].

## Results

Between January 2021 and May 2022, 21 patients were interviewed. Twelve participants were women, and the mean age was 85.2 years (SD 6.45). Table 1 summarizes participant characteristics.

**Table 1:** Patient characteristics

Number of patients *n* = 21		*n* (%)
Sex	Female	*n* = 12 (57%)
	Male	*n* = 9 (43%)
Age range	70–75	*n* = 3 (14%)
	76–80	*n* = 2 (9%)
	81–85	*n* = 4 (19%)
	86–90	*n* = 8 (38%)
	91–95	*n* = 4 (19%)
Injury	Hip fracture	*n* = 15 (71%)
	Hip contusion	*n* = 2 (9%)
	Radius fracture	*n* = 1 (5%)
	Humerus fracture	*n* = 1 (5%)
	Spinal fracture	*n* = 1 (5%)
	Elbow dislocation	*n* = 1 (5%)
	Pubic fracture	*n* = 1 (5%)
Cognitively impaired^1^	Yes	*n* = 7 (34%)
	No	*n* = 14 (66%)
Preinjury functioning	Independently	*n* = 6 (29%)
	Walking aid, adequate activity^2^	*n* = 7 (33%)
	Walking aid, limited activity^3^	*n* = 7 (33%)
	Wheelchair bound	*n* = 1 (5%)
Living arrangements	Care facility	*n* = 3 (14%)
	Spouse and/or family	*n* = 9 (43%)
	Alone	*n* = 9 (43%)

^1^based on clinical assessment by a doctor

^2^able to perform ADL activities independently or with limited help from a community nurse/family member

^3^dependent on the community for ADL activities; unable to leave the house without help

During the interviews, participants shared insights into their GTU stay, and discussed their perspectives, recovery priorities, and expectations of the GTU’s environment and staff. Patients likened their experiences to a care journey, leading to the emergence of four key interconnected themes: (1) patients in the backseat, (2) patients in the passenger seat, (3) patients’ luggage, and (4) the route planner, each with two to three subthemes (Table 2). Although these themes were not addressed as separate issues during the interviews, the findings are presented under four headings to reflect variations in each step of the journey.

**Table 2:** Summary of the identified themes

Theme	Description	Subthemes
1. Patients in the backseat	The hospitalization experience can prompt a profound transformation in the self-perception of older patients, stemming from a sudden decline in physical and, at times, mental well-being. Consequently, hospitalization accentuates the focus on aging and its associated challenges. The rapid and drastic nature of these changes leaves individuals feeling vulnerable, lacking control and occasionally gripped by fear. Moreover, the necessity of relinquishing physical independence following fall-related injuries initiates a shift in one’s familial and societal roles. Older patients transition from being caregivers to care recipients, often finding themselves relegated to a less active role—taking a backseat, figuratively speaking.	1. Anxiety and experiences of loss 2. Guilt
2. Patients in the passenger seat	Navigating the care pathway for older patients recovering from fall-related injuries often proves to be a complex and intimidating journey. While these individuals typically have full autonomy over their lives pre-hospitalization—akin to sitting in the driver’s seat—their roles shift to a more passive stance, resembling that of a backseat passenger, upon entering the healthcare system due to declining health and the uncertainties surrounding their condition. Despite this transition, values such as independence and personal autonomy maintain significant importance for older patients. The strong desire to uphold this independence usually remains a key priority. However, these wishes are frequently left unspoken by older patients and can be inadvertently undermined. Actions such as offering assistance beyond physical needs or making decisions in conjunction with family members instead of providing choices regarding their care can inadvertently nurture dependence in these individuals, compounding feelings of lost control. Interestingly, concerning clinical decision-making, older patients may willingly cede some control, allowing family members or healthcare providers to take the lead. In this context, the need for self-determination finds balance as patients actively choose not to assert control, thereby occupying the “shotgun seat” where they guide decisions without direct operation—a form of informed decision-making.	1. Independence 2.Personal Autonomy
3. Patients’ luggage	Norms and values tint the expectations related to being admitted to the hospital and shape how older patients evaluate the care they receive as well as the healthcare professionals delivering that care. Furthermore, ideas that older patients have about embodying the concept of being a “good” patient impact the interactions between patients and healthcare providers. As a result, the preexisting attitudes and beliefs that patients carry with them to the hospital (i.e., their luggage) significantly influence the overall experience.	1. Being a patient 2. healthcare professional identity
4. The route planner	The moment an older patient with a fall-related injury enters the emergency department, the GTU care pathway springs into action; one of its goals is to admit the patient in the GTU as quickly as possible. This is achieved by working with standard order sets that match the integrated care-plan each step of the way. The theme ‘the route planner’ maps patient journey in the acute care setting and describes how older patients experienced the coordination of care, (interprofessional) communication and the hospital facilities overall and the geriatric trauma unit in particular	1. The care pathway 2. The ward 3. The team

### Patients in the back seat

Upon entering the hospital setting, patients often find themselves reluctantly surrendering control to healthcare professionals (HPs). While they recognize the prioritization of expected health benefits over personal autonomy, this transfer of control is frequently accompanied by emotions of vulnerability, distress, guilt and a perception of reduced self-worth.

#### Anxiety and experiences of loss

While the majority of participants maintained an optimistic outlook on their future functional abilities and exhibited forward-thinking attitudes, instances of anxiety emerged in certain situations. These concerns ranged from unfamiliarity with hospital routines, and uncertainties regarding discharge planning, to challenges performing daily routines outside of the hospital environment such as walking one’s dog. The nature of these apprehensions was significantly influenced by the stage of their hospital stay. Participants in the early post-surgery phase expressed worries about regaining their functional capacities, while those further along in their recovery process fretted over the long-term consequences on their activities such as biking, independent shopping, or living autonomously. The abrupt onset of physical and occasionally cognitive decline prompted a shift in self-perception.

#### Guilt

Guilt was a prominent theme throughout the interviews, particularly in discussions related to familial support dynamics. Participants delineated both the practical and emotional support they provided, encompassing practical tasks such as laundry, to care coordination. Despite the appreciation for these roles, a prevailing sense of guilt was evident among participants regarding their reliance on informal caregivers. For instance, quite a few respondents highlighted the inherent tension in soliciting assistance from children who are also managing their personal and familial commitments.

To mitigate the burden on their informal caregivers, some participants made healthcare decisions prioritizing the logistical convenience of their families over their own health preferences, such as selecting rehabilitation facilities based on proximity rather than the specialized services offered.

The complexity deepened for those also acting as caregivers for their spouses, resulting in a compounded sense of inadequacy. They grappled with the dual challenges of not fully meeting the partners’ emotional and physical needs while also imposing additional care responsibilities on their children. This quote illuminates the intricate balance between personal health needs and familial bonds:

*“I believe that being with my husband is more important than my own rehabilitation. If I go to that rehabilitation home then, of course, I have a different physical therapist than here; he can only give therapy three times a week. Here, they thought that was unfortunate, but on the other hand, do I have to be separated from my husband for that? So I am here and my husband over there? He cannot handle that. Besides, you can exercise by yourself.” -Participant 6*.

### Patients in the passenger seat

The experience of hospitalization following falls signifies an abrupt departure from autonomy into a realm characterized by chaos and uncertainty. Participants find themselves thrust into a state of dependency, relying on others for support and assistance. This shift prompts a profound re-evaluation of roles, as individuals strive to reclaim agency with the aid of their support network. Consequently, they move from a position of passivity to one of active engagement, metaphorically transitioning from the backseat to the passenger seat of their own lives.

*“We suddenly became dependent on our children while we were independent. 100% independent.’’ -Participant 10*.

#### Independence

Participants perceived ‘independence’ as the ability to perform physical tasks unaided. For all participants, maintaining independence held paramount importance. The extent of independence participants aimed for was contingent to their preinjury functional status. Despite all participants having specific goals, such as dressing unaided or walking stairs, they often refrained from communicating them to HPs, assuming their goals to be self-evident.

*‘’Yes, well… I think it’s obvious. ’You don’t want to stay in this situation’’ –Participant 6*.

Despite physical challenges, most participants remained optimistic about returning to preinjury functioning with determination and support. They perceived restoring and maintaining function as personal responsibility, acknowledging the supportive role of HPs, but emphasizing their own investment of effort in the process:

*“I do everything [the exercises given by the physiotherapist] as best I can, because the doctor said I shouldn’t force it. However, I try as much as possible, I really do. This morning in bed when the nurses were busy, I did the exercises right away [demonstrates]… you know, with my arms like so— and a little bit with the shoulders and so…. Yes, I’m doing my best, but it takes effort.” -participant 1*.

Their ability to focus on progress and positive aspects underscores resilience and a forward-thinking approach amid potential postinjury limitations. Even participants dealing with chronic illnesses refrained from identifying themselves as being ill.

*‘’At this age you no longer have goals. No, I lived my life as a queen. I always say, I am blissfully content without any wishes.First of all, my children are healthy. That is most important to me. My grandchildren are healthy. And I don’t have any ailments. -Participant 12 (suffering from hip fracture and chronic heart failure)*.

#### Personal autonomy

Most participants perceived control not only as having the physical ability to perform tasks independently but also as the authority to determine the necessary care and how it should be administered. This notion of control, encapsulated in personal autonomy, denotes the freedom and capacity to act on one’s own behalf, extending beyond self-resilience to include decision-making powers. While factors, such as a rigid schedule and unfamiliar surroundings, posed constraints on personal autonomy, older patients employed strategies to reestablish a degree of influence. These tactics involved asserting themselves, forming partnerships with HPs, and seeking support from family.

*Assertiveness.* Assertiveness is often used as tactic in interactions between participants and HPs during activities of daily living (ADL). As regaining independence was of paramount importance, all participants actively sought to manage their own needs whenever feasible. When decisions were made by HPs, patients were not always given the opportunity to voice their preferences. However, when participants voiced their wishes explicitly, these were typically respected and accommodated.

*“[When the nurse started taking control] I said,“no, don’t take over. I can stand and take a shower, I’ll be fine.” Then, the nurse observed and assisted me on my terms.” -Participant 7*.

This was also observed when patients actively asked for accurate and timely information about their situation/condition. As one participant noted:

*“Afterwards, the explanations improved because I started asking more questions. The nurses did not spontaneously explain what was happening.” -Participant 5*.

In this context, participants established control by actively engaging in their care. However, personal autonomy can also be realized by abstaining from involvement. In terms of medical decision-making, the majority of participants deliberately opted to relinquish their control to family members or HPs. This exchange in control was often underpinned by a strong sense of trust in the expertise and intentions of both family and HPs.

*“All decisions are consulted with my children. The children do what is right for me, as I used to do for them.” -Participant 3*.

And

*“I assume that those who help you are skilled because it is a hospital. That is a resignation of trust.” -Participant 18*.

The timing of decision-making emerged as a critical factor, particularly in high-stress environments such as emergency departments (ED) or perioperative periods. In these contexts, participants sometimes found themselves involuntarily surrendering control due to the overwhelming nature of the circumstances, resulting in a sense of disempowerment.

*Partnership.* Participants strategically engaged in partnership-building with HPs as a means to reinforce their personal autonomy. This practice involved initiating conversations with the aim of discovering common interests and establishing personal connections with HPs. By fostering a sense of shared humanity and trust through these partnerships, participants found it more manageable to relinquish control to HPs. However, effective partnerships require mutual effort; HPs must also invest in understanding their patients. In discussing expectations, participants highlighted values such as equality, thoughtfulness, openness, and warmth as crucial factors in care provision. These elements not only enhance personal autonomy but also contribute to a sense of security. Yet, a nuanced perspective emerged in which HPs were perceived as both helpful and considerate but also challenging to approach due to the busyness of the ward. This often led to superficial interactions that hindered the development of meaningful relationships.

*“They [the nurses] do listen to you when you’re in pain, but it is not a heart-to-heart or anything.” -Participant 4*.

Participants valued HPs’ understanding, positive reinforcement, and coaching as supportive elements in their recovery, fostering trust and motivation. While humor plays a dual role in easing tension and fostering motivation, it sometimes poses a barrier to discussing deeper emotions and concerns.

*“You don’t want to show the back of your hand right away, and when they [the nurses] are very cheerful, you will go along with that. You really need someone who dares to start a real conversation, then you will open up and emotions will run free. […] For that to happen you need time and space… and the right HP.” -Participant 10*.

The receptiveness of HPs varied within GTU teams, with some cultivating close connections with participants while others maintained a more formal and transactional approach in their interactions.

*Family.* Almost all participants relied on their family for support during the journey with fall-related injury, both on a practical level, such as household tasks, and on a mental and emotional level. Family members not only assisted in making medical decisions on the participants’ behalf, but also played a crucial role in overcoming communication barriers and facilitating the exchange of information.

*‘’[…] My sister always accompanies me to appointments and knows everything. […] I have a hard time remembering what was said, but she always remembers. Therefore, I prefer it if they [HPs] discuss things with my sister. She’ll tell me”-Participant 9*.

For many participants, their children serve as a primary source of support. In cases where participants did not have children, a partner or other family member, like a niece or nephew, assumed a supportive role. The reliance on family members is widely acknowledged by HPs.

### Patient luggage

Norms, values and prior healthcare encounters significantly influenced how participants interpreted their hospitalization experiences, acting as metaphorical luggage that, despite its uniqueness to each individual, revealed two prevalent attitudes shaped by shared societal norms and internalized values. These encompassed participants’ self-perceptions as patients and their perspectives regarding HPs.

#### Self-perception of being a patient

All participants belong to a generation traditionally inclined towards a passive patient role, characterized not by lack of engagement but by a deeply ingrained respect and trust in authoritative figures within the healthcare system. This passive stance is not synonymous with inactivity, but is rooted in cultural values that prioritized deference to medical authority.

Participants’ reflections on their hospital experiences were often positive, emphasizing contentment and gratitude for the care received. Even when expressing criticism, participants often contextualized their concerns, attributing any care-related discrepancies to their own inadequacies:

*“Well, sometimes I have to wait and I don’t like it. However, I would also despise having to care for moaning people. Stop pitying yourself! Old folks like me tend to do this…” -Participant 20*.

Despite the growing emphasis on active participation and shared decision-making in healthcare, participants typically did not adopt assertiveness as a strategy for assuring personal autonomy. Participation was seen as an opportunity rather than an inherent right. However, since most participants wanted to participate in their care, they employed strategies to maneuver between compliance and asserting agency. Whether through humor or initiating components of their care autonomously, participants sought to balance deference with active participation in their healthcare journey.

*“Did you get help getting out of bed?” -Interviewer*.

*“I would have if I’d asked… However, I do a lot by myself. I like to be involved in my recovery because it fosters progress. […] this morning I had physiotherapy. As I knew what time he [the physiotherapist] be in, I tried to position myself on the edge [of the bed] upon his arrival.” -Participant 4*.

Initially, acceptance dominated -the acknowledgement of the situation to facilitate a smoother transition toward recovery. Yet, the majority of participants later adopted a more autonomous approach during hospitalization. However, a subset of participants did not manage to do so, leading them to transition to a state of submission or resignation. A common feature of this group was their lack of a positive outlook on recovery, in contrast to the perspectives of other participants.

*“They’ve [HPs] taken over, because I’m unable to do anything on my own.” -Participant 11*.

and

*“It just happened to me, I had no say in the matter. Therefore I’ll simply leave it [the care and control] up to them [HPs]. -Participant 19*.

This passive behavior may stem from feelings of vulnerability and a perceived lack of control over one’s situation. The divergent responses to hospitalization ranging from resilience and active reassertment of independence to resignation, highlight the complex interplay between individual dispositions, cultural norms and the healthcare environment.

#### HPs’ professional identity

The professional identity of HPs as perceived by participants plays a crucial role in determining the dynamics of patient-provider interactions, particularly in relation to nurses. Patients frequently leaned on stereotypes and ingrained biases when assessing HPs, a phenomenon that shaped their hospital experiences and set their expectations.

In patient perceptions, doctors often embodied a portrayal of mature, imposing figures, leading to moments of perplexity when confronted with younger or female physicians. These encounters reveal the gap between patient expectations and the evolving demographics of the medical profession.

*“[…] Then two young men came in and I didn’t realize that they were doctors.–Participant 12*.

Conversely, nurses were frequently characterized by traits such as femininity, youthfulness and kindness, with a pronounced emphasis on their nurturing disposition. Notably absent from these descriptions were attributes such as expertise or professionalism; relegating the role of nursing primarily to the realm of physical caregiving. Patient narratives often revolved around tasks assisting with ADL and fulfilling basic needs, overshadowing the recognition of nurses as independent healthcare professionals with specialized knowledge.

*“I haven’t received a lot of information from nurses because, well, that’s not their job. The nurses are here to take [physical] care of me.” -pPrticipant 13*.

Being perceived as assistants to doctors led to the majority of participants refraining from confiding in nurses, reserving their questions for other professionals.

*“I thought I should ask the doctor why that [the dosage of the medication] changed, but I have not seen him in the past two days. So I took the pills anyway.” -Participant 1*.

*“Why haven’t you asked the nurses for advice?” -Interviewer*.

*“Well, I don’t believe their knowledge reaches that far.” -Participant 1*.

Such perceptions permeate participants’ views of nurses as supplementary to doctors rather than as integral members of the healthcare team.

Few participants felt inclined or capable discussing their anxieties with nurses. Reasons for this reluctance varied; some participants believed nurses were occupied and lacked the ability to provide emotional support, while others considered it inappropriate to share fears with nurses, preferring to confide in family instead. This perspective potentially reflects a broader sentiment among participants that emotional support forms a peripheral rather than a core component of nursing.

### The route planner

Upon arrival at the ED, older patients with fall-induced injuries trigger the GTU care pathway, with a primary goal of swift admission to the GTU. This is accomplished through the utilization of standardized order sets that align with a comprehensive care plan at each stage. The theme ‘the route planner’ encapsulated patients’ perceptions and experiences navigating their hospitalization journey.

#### The care pathway

While a majority of participants percieved the transition from ED to the GTU as streamlined and efficient, only four could accurately recall the intricate details of their patient journey. For many, the period spanning from admission through postoperative recovery was shrouded in ambiguity, engendering a sense of bewilderment regarding the operational intricacies of their care experience:

*“I had absolutely no insight into the organization. I had no idea what everyone was doing and what they were going to do with me” -Participant 5*.

*Waiting.* A noteworthy subtheme that emerged consistently in the data were participants’ collective perception of the care pathway as disjointed rather than a cohesive journey from start to finish. This led to a continual sense of awaiting the next phase in the recovery process. One aptly articulated this sentiment, stating:

*“An occasional walk or so, but what else must I do? Nothing, nothing at all. It’s [patient’s recovery] being reviewed daily […] I was supposed to leave yesterday. Uhm no, not yesterday but today. I was supposed to go home today. However, this morning they said, ‘just wait another day.” -Participant 16*.

In the ED, participants had to await approval to be included in the GTU pathway (where both the surgeon and geriatrician had to concur that the patient met the criteria). Subsequently, a further delay ensued for surgery, succeeded by the commencement of the process of regaining mobility. Participants believed that true recovery did not commence immediately after surgery but rather upon admission to a rehabilitation center. While most participants felt frustrated by these delays, they tended to keep these feelings for themselves rather than discussing them with HPs.

*Information.* Participants encountered varied experiences regarding information dissemination. Some could recall treatment details from the ED, but had gaps in their memory about interactions with HPs during hospitalization. Many participants struggled to pinpoint sources and timing of information provision throughout their stay, suggesting issues with retention rather than comprehension. Visual aids alongside verbal instructions significantly enhanced information retention compared to verbal instructions alone.

All participants needed organized discharge planning, which was initiated shortly after admission. While preferences for rehabilitation were commonly sought, participants were often excluded from the planning process. HPs often reach out to family members to finalize arrangements. Although this approach generally met with acceptance, some participants desired more proactive information sharing from HPs regarding their care and treatment plans.

#### The ward

All participants adhered to the routine of the GTU and the expectations of HPs regarding (early) mobilization. They understood early mobilization as a means of regaining their preinjury level of function rather than as a strategy to prevent complications. Participants’ focus on physical recovery likely contributed to the better retention of information provided by physiotherapists among most patients. Furthermore, a considerable number of participants remarked on the encouraging manner in which physiotherapists engaged with them. Nurses’ actions, such as assisting patients in mobilizing and engaging in communal activities, and promoting the use of regular clothing, were valued. However, these actions were not perceived as rehabilitative efforts by participants, nor were they explained as such.

Although all participants acknowledged receiving assistance with ADL, the GTU’s goals of fostering self-care and preserving personal autonomy were inconsistent. Many participants felt that the focus on patient participation extended only minimally beyond washing one’s face. While most participants aspired to actively participate in their care rather than being solely reliant on others, they often hesitated to assert their preferences within the established unit routine. There was a perceived reluctance to disrupt routine or voice individual preferences.

The majority of participants were content with the GTU’s environment, describing it as clean and pleasant, with effective wayfinding. The presence of the GTU lounge and its activity counsellor was regarded as a valuable asset by a considerable number of participants, providing space for socializing and mental engagement.

*“I went to the living room yesterday, it was very nice. I spent some time talking to a lady, I think she’s in charge […] yes, having an actual conversation was great.” -Participant 7*.

However, the GTU lounge, unique as it is, failed to be routinely highlighted by HPs as part of the patient experience. The consistent guidance of participants with cognitive impairments to the lounge, a move seemingly geared toward providing support and aiding in their special orientation within the unit, is noteworthy.

All participants felt that their desire for privacy was consistently met, with many expressing a preference for shared accommodations over single rooms due to the social camaraderie it facilitated.

*“Here [double room], I can bring the people sitting in a wheelchair a nice cup of coffee by putting the cup on my walker. This way I can help and stay in contact with others.” -Participant 3*.

#### The team

The GTU team consists of doctors, (geriatric and surgery) nurses, nurse assistants, a dietary care specialist, physiotherapists, discharge planners and a social worker. These professionals interacted with patients on an almost daily basis. However, many participants struggled to differentiate between doctors and nurses, and other professionals. It is unclear if this was because HPs did not introduce themselves or because participants could not remember.

Most participants reported positive team cohesion within the GTU, noting a high level of camaraderie among HPs who demonstrated a thorough understanding of patient circumstances. Importantly, participants praised the team’s ability to maintain consistency in their messaging, eliminating the need for repetitive information sharing. This contributed to an overall perception of professionalism within the unit.

However, participants also discerned varied attitudes among HPs. While many were commended for their kindness and willingness to invest time in patients, a subset was noted for their tendency to dismiss complaints and exhibit impatience, particularly toward slower-paced individuals. This differential approach was seen as a counterproductive way to foster collaborative and constructive patient-provider relationships.

*“One [nurse] says: “if you have to pee that much then you shouldn’t drink so much.” And then you get another nurse and she says: “you should drink more…” can you imagine? And then I have to go to the toilet […] and the nurse helping me sighs… I retort by saying “well, I can’t put a cork in it.”, but she says nothing in return. She has no humor… Well, let’s say some [nurses] are more willing to help than others.” -Participant 3*.

## Discussion

The objective of this study was to investigate how patients perceived their stay in a GTU and to identify whether the GTU’s stated focus on PCC was evident in these interactions. The journey following a fall-related injury was found to be complex and challenging for older patients, signifying a loss of independence and personal autonomy. Given the GTU’s explicit commitment to PCC and an adept interprofessional team specializing in geriatric care, an expectation was set to uncover examples of HPs going beyond the conventional injury-oriented perspective and truly engaging with patients, fostering a milieu conducive to open discussions about anxieties and needs. However, findings revealed a disparity in consistently and continuously implementing PCC, with a majority of HPs continuing to perceive patients primarily in terms of their injuries rather than as individuals with unique healthcare needs. While concentrating on fracture-specific aspects of care and physical rehabilitation mirrors some patient preferences, neglecting broader needs such as patient involvement in care (decisions), the emotional burden of the fall-related injury, and the fundamental need for (regaining) personal autonomy, compromises the holistic approach to care within the GTU.

In their review, Kitson et al. [40] delineated three fundamental elements integral to PCC, namely patient participation and involvement, the patient-provider relationship, and the care environment. While the latter aligns with patient preferences of regaining independence, deficits were observed in the realms of patient participation and the patient-provider interplay.

The element patient participation and involvement amalgamates three aspects of care: patients are respected as autonomous individuals, care plans are tailored to individual needs and care addresses physical and emotional needs. All patients advocated for active involvement in their care, acknowledging the obligation to restore and sustain personal autonomy and independence as a personal responsibility. The emphasis on participation in physical care, underscores the perceived insufficiency in current patient engagement levels, despite the desire for greater involvement. Patient participation is a steadfast component of PCC, signifying its importance. For the successful execution of a PCC paradigm, patient participation is an indispensable and non-negotiable element [38, 40]. Despite patients expressing a desire for control over physical aspects of care, the majority did not exhibit active interest in engaging in formal care processes, such as clinical decision-making or discharge planning. Participation, according to patients, meant *information*, which is in line with previous studies [39, 52–56]. Patients sought transparency regarding their care journey and placed value on being actively listened to when articulating their concerns; however, they generally deferred clinical decision-making to HPs and/or family. While this might be interpreted as loss of control or as passive behavior, patients did not interpret it as such when met with attentive and empathetic responses from HPs; the need for self-determination was fulfilled by deciding *not* to exercise control and instead trusting the judgment of staff or family. While shared decision-making is a cornerstone of PCC [40], it is vital to recognize that barriers such as perceived knowledge gaps, low self-efficacy, anxiety, and reticence toward seeking clarifications can impede patient participation. Acknowledging the diverse preferences among older patients regarding their involvement in care planning is imperative due to the variability in individual needs [57–60]. HPs can navigate these hurdles by proactively encouraging patient inquiries, tailoring information to their cognitive capabilities, and proactively asking patients if and to what extent they themselves or their family want to be involved in clinical decision-making [60]. Patients were generally positive about their recovery, yet moments of fear and anxiety surfaced. The specific concerns that preoccupied patients were notably contingent on the phase of hospitalization they were navigating. Following surgery, individuals tended to direct their apprehensions toward physical recuperation. Conversely, conversations held in later stages of recovery often saw a shift in focus toward apprehensions concerning future implications and the impact of their injury on interpersonal relationships. Mental well-being gained significance as patients approached the end of their hospital stay. However, unlike physical recovery, little attention has been given to this aspect of care [52, 56]. The concept of evolving patient needs throughout the hospitalization process resonates with findings from Jacelon et al.‘s [61] study on autonomy transitions, underscoring the necessity for HPs to recognize and respond to these transitional phases effectively. In addition to professional support, older patients acknowledged the need for extra help from family or friends during their recovery process. While most patients were glad with the support they received, help came at a price-dependence of older patients, causing strain on their already burdened children, which amplified feelings of guilt and worry. This emotional turbulence, intricately intertwined with compromised morale and experiences of powerlessness, underscores the significance of equitable and reciprocal relationships within the caregiving dynamic [56, 62–64]. Patients’ inclinations toward actions that prioritize caregiver convenience over personal needs, although momentarily easing familial burdens, may inadvertently impede the attainment of long-term recovery and sustained independence.

The significance of the second element of PCC, the patient-provider relationship, was prominently illustrated by our findings. Older patients appear to value human connectedness more than other issues, and their overall care experience is influenced by interpersonal interactions. According to Richards et al. [65], PCC is contingent upon fostering a culture and dialogue that centers around the patient. Core to this approach are proximity, mutual respect, and openness. Although participants acknowledged the kindness and professionalism of HPs and their dedication to ‘getting the job done’, they felt that these qualities were often overrun by the formalities of care, such as discharge protocols, the ward’s busyness, and a procedural focus that sidelined patient narrative. This led us to infer that the notion of partnership was missing in the GTU. Patients’ perspectives highlight the importance of partnerships. Knowing the patient’s ‘life-luggage’, by empathic listening, humor and discussions about personal interests, finds common ground to start a collaborative relationship with room to discuss preferences and patient participation in care [66, 67]. Besides attempting to make care more patient-centered by investing in a patient-provider relationship, recognizing that partnership is influenced by the dynamics through which individuals’ behaviors, actions, and interactions are molded by internalized values, societal norms, and cultural influences is fundamental. Patients’ reluctance to question care being overtaken might stem from past experiences or cultural influence toward compliance [68]; just as HPs’ communication styles are influenced by their training and organizational norms. Recognizing and understanding these constructed roles is crucial for fostering effective communication, building trust and enhancing patient outcomes [40, 66, 69].

The third element to PCC, the context where care was delivered, outlines what is needed in the healthcare environment to deliver PCC. Patients’ primary goal while hospitalized was to regain their independence as quickly as possible. As independence is related to preinjury functioning, patients predominantly focused on physical recovery during hospitalization [54, 70–72]. Since the GTU’s environment is tailored to enhance functional recovery, it was met with patients’ approval. The presence of a space for socializing and mental engagement was also regarded as a valuable asset of the GTU. Furthermore, interprofessional communication was perceived as efficient. Patients generally received a consistent set of messages and found staff knowledgeable about their personal situation. Despite these positives, patients felt that care activities within the GTU were fragmented rather than seamlessly integrated. This fragmentation became evident in patients’ understanding – or lack thereof– of how various HPs’ efforts, such as nurses assisting with ADL and physiotherapists overseeing exercises, aimed at achieving a shared goal of enhancing personal autonomy and independence. This perceived fragmentation occasionally left patients feeling that their recovery was impaired due to time constraints or the absence of the appropriate professional for certain tasks, underscoring moments of reduced control and the static but unpredictable GTU schedule.

The intention of integrating PCC into standardized care is an important and valuable characteristic of the GTU. However, the process of consistently and continuously implementing PCC is arduous and complex. While the GTU environment aligns well with patients’ preferences for physical rehabilitation, the concentration on the fracture-specific aspects of care undermines a more holistic approach. The identified themes indicate that participation in the physical aspects of care are of great importance to older patients and that partnership is needed to overcome the challenges of fall-related injury. Given the partial adoption of key PCC principles within the GTU, it is unsurprising that the findings keep with previous literature, such as limited patient participation [54, 56, 71, 73], insufficient psychological support [74, 75], participation in the formal aspects of care [66, 76], and a perceived lack of control during hospitalization [56, 75].

### Strengths and limitations

This study has some limitations. The COVID-19 pandemic resulted in visitor restriction policies that limited our ability to collect data for 6 months. This could have negatively affected patients’ experience with patient and family participation in the GTU. Second, while this study was conducted in three GTUs across the Netherlands, findings may not be one-on-one transferable to other care settings, institutions or countries because slight variations exist in the way GTU care is conducted. For example, just one out of three hospitals had an activity counsellor working in their lounge. While the variations are small and do not infringe on GTU principles, they can make the transferability of the results difficult. Nevertheless, qualitative studies, such as this, provide a platform for older patients to speak their mind and elucidate what is important to them. This provides valuable insight into the workings of the GTU model from a patient’s point of view, which can be used to review the GTU care pathway and guide future studies. Moreover, an effort was made to increase the representativeness of participants by including patients who were diagnosed with cognitive impairment [77]. As this is a growing group, we believed it necessary to capture their perspective, because they are often excluded from studies for practical reasons. Patients with memory loss experienced their care and recovery process much the same as other older patients—they found it important to feel supported and wanted a social connection with others. The only difference found was the need for a social space. Where other patients did not mind sitting in their patient room, patients with cognitive impairment appreciated the GTU lounge and its activity counsellor. It provided them the opportunity to perform familiar and enjoyable activities to keep their brain and body active. Spending time in the GTU lounge gave patients with memory loss a sense of purpose.

### Implications

Our findings suggest that successful implementation of PCC in healthcare requires adherence to all its core elements. This necessitates a shared understanding of PCC throughout the interdisciplinary team to ensure its integration in the care pathway. HPs must acknowledge the equal importance of patient participation and the patient-provider relationship alongside a tailored care environment.

This research highlights the significance of establishing a strong sense of human connection for patients’ well-being, as patients are more likely to engage and communicate effectively in its presence. HPs play a crucial role in fostering this relationship by dedicating time to meaningful conversations that prioritize patient needs and concerns. Recognizing and acting upon these subtleties offer avenues for tailored patient participation, enhancing overall recovery outcomes by addressing issues that are vital to patients.

It is essential for HPs to value their soft skills—such as kindness, respect, compassion, and empathy—just as much as clinical expertise and efficiency. Promoting and enhancing these interpersonal skills through training and feedback can lay a solid foundation for PCC and should be emphasized across healthcare systems.

## Conclusions

This study highlights the complexities and challenges inherent in delivering PCC within GTU’s. Despite the GTU’s commitment to PCC and its specialized interprofessional team, the actual implementation of patient-centered approaches often fell short of expectations. The findings emphasize the need for consistent and holistic application of PCC principles that go beyond mere physical rehabilitation to include emotional support, patient involvement in decision-making, and the fostering of autonomy. To truly embody the ethos of PCC, it is crucial for HPs to treat patients not just as cases to be managed, but as individuals with unique needs and preferences. Moving forward, the integration of PCC into standardized care must address these gaps to enhance both the quality of care and patient satisfaction within the GTU setting.

### Electronic supplementary material

Below is the link to the electronic supplementary material.

Supplementary Material 1

Supplementary Material 2

Supplementary Material 3

Supplementary Material 4

## Data Availability

All data generated or analyzed during this study are included in this published article and its supplementary information files.

## Abbreviations

GTU: Geriatric Trauma Unit
PCC: Patient-Centered Care
HP(s): Healthcare professional(s)
ED: Emergency department
