# Supplementary material for: Exploring geriatric trauma unit experiences through patients’ eyes: a qualitative study
Source: BMC Geriatr. 2024 May 30;24:476. doi: 10.1186/s12877-024-05023-z (PMC11140891; doi:10.1186/s12877-024-05023-z)
Supplement: Supplementary file 1 — Supplementary Material 1 [file 12877_2024_5023_MOESM1_ESM.docx]

Additional file 1

**Quotation table**
*This table compiles all quotes from the research, categorized according to the themes and codes identified in the initial phase of analysis. It serves as a comprehensive reference, providing a structured overview of the qualitative data, facilitating a deeper understanding of the patterns and insights derived from the study. Side note, the quotes may vary slightly from the original due to the translation from Dutch to English.*

| **Theme 1 – Patients in the backseat** | | | |
| --- | --- | --- | --- |
|  | **1. Anxiety and experiences of loss** | Patients, who were autonomous prior to admittance, had to suspend their autonomy by relying on the hospital and its staff for treatment. The situation they ended up in, is new and ultimately frightening, due to the alien environment, the fast pace in hospital and the isolation. Older patients cannot fully grasp what is happening and what will come, because it is unpredictable. Will they be able to return home and function like they did before the accident? Not knowing emphasizes the feeling of loss of control. | |
|  | Subcode 1: **Feelings about hospital admittance** | As a patient I found the whole situation challenging. I was constantly wondering what the next step would be  Interviewer: Yeah.  And I'm concerned about whether they take me seriously.  Interviewer: Yes.  Um, it was more about the way they communicated, you know, the tone...  Interviewer: So it was about the tone?  Yeah. It felt like they were saying, "we know it all and that bothered me.  Interviewer: Yes. | Participant 5 |
|  |  | But again, they did their best and um... Look, if you've never been in a hospital before… it was my first time. In the middle of the night I heard all sorts of sounds. I got nervous and thought ‘oh, what are they going to do…’ | Participant 5 |
|  |  | Let's be honest, as a guy, that's a bit tough to swallow [receiving help showering from a very young, female nurse]. | Participant 5 |
|  |  | Yes, and also um... that others are involved with you and indeed, what's happening and um... Indeed, uncertainty, what are they going to do? | Participant 5 |
|  |  | But once I was lying there [on the operating table]... that was the moment I started thinking about the consequences of the fall... | Participant 5 |
|  |  | Yes...I was a bit confused [the patient went through a delirium] ... it's a very unpleasant feeling... I kept thinking... I hope they don't put me in a mental institution... And then they didn't take what I said seriously... it was disagreeable. | Participant 7 |
|  |  | [..] it was during one of the first nights... I remember thinking, where on earth am I now? And, well… I hope I won't be staying here for long... I still don't know where I'm heading after discharge…  Interviewer: No... does that also cause tension?  Yes, it really does... it’s truly unsetting. | Participant 6 |
|  |  | When they take you away, what's that process like? Those kinds of things really stress me out. Right now, I can just pass on responsibilities. Could you check my schedule? Give a heads-up to the pedicurist; otherwise, I'll be stuck with the bill. And make a call to general practitioners’ office, let them know I won't be able to make it.  Interviewer: You've got someone to rely on now.  Yeah, quite a bit. But, you know, that person is only human; they face challenges too.  Interviewer: Absolutely. Would you share these kinds of feelings and things with, say, the nurses?  No, you don't share that with the staff. I also think that if I didn't have someone to rely on, the doctor would probably say, "put him in a nursing home and let him spend his last years there, and that's something I really don't want. | Participant 8 |
|  |  | There was a lot of uncertainty, which I experienced as a setback, and honestly, it’s…. yes, the actually hit me, what do I still have left? | Participant 10 |
|  |  | That's after the first week. Initially, everyone is still concerned, and the kids come by. Then, one day, they skip a visit. I find myself pacing, and that's when you feel alone here. | Participant 10 |
|  |  | I had never been washed by a young woman, a nurse. That's also something when you're so full of life and suddenly confronted with that. | Participant 10 |
|  |  | That's when your anxiety starts kicking in, you know. | Participant 12 |
|  |  | Yeah, that's why I'm waiting until I can be discharged. It can't be too soon because they also mentioned, if you leave earlier, it's not as easy to come back in case things aren't going well. | Participant 20 |
|  |  | And if needed, one of the male nurses, he washes my back.  Interviewer: Yes  And he washes everything. And I'm not shy about it. The male nurses can wash people too. | Participant 14 |
|  |  | Interviewer: Yeah. And, um, does it feel different for you because, initially, you could, you know, do it yourself? Like, do you get the sense of having to wait because, under normal circumstances, you would handle it on your own?  Yeah, quite a bit, yeah. | Participant 13 |
|  |  | When you get sick, then you – to some degree – know what to expect. At some point you’ll get sicker and then you need to go to the hospital. However, now… It’s liked pulled a trigger and here you are. The suddenness of it all… You first think ‘what happened?’ That’s in the first week. You receive attention and the kids come to visit… then, someday, nobody comes and you sit there and all of a sudden it [the emotions] become intense. | Participant 12 |
|  |  | If I had no one to support me the doctor would have probably said, ‘put him in an old folk home so he can spend his last years there.’ That’s something I truly don’t want because then I’ll lose all my contacts here in the street. That’s no life for me | Participant 8 |
|  | Subcode 2: **feelings towards the fall** | […] Well, that also depends on me.... Because I feel quite beaten down.... | Participant 5 |
|  |  | Interviewer: What are you worried about?  That I.., that I become dependent on someone..... But I'm afraid.. you know what I mean.. | Participant 5 |
|  |  | […] Because I feel like I don't have a life... Every day, I have to stay at home. I've been stuck at home for nine weeks, unable to leave the house. When the nurses come, you have to welcome them, I've had a proper upbringing. But then, showing them out is a struggle for me [because the patient had difficulties walking]. | Participant 7 |
|  |  | [silence] One thing I'm very sure about. As you age, you reach a certain point where... at any moment, you could die... anything can happen, and you should just accept that. It's not that I'm looking forward to it, but I can imagine that it could happen, yeah. | Participant 1 |
|  |  | Overall, yes. I haven't thought about it terribly much, but overall, I do. You know, as I get older, just getting older and older... eventually, I won't be here anymore, and I find that quite difficult to accept. | Participant 1 |
|  |  | I actually feel hopeless since the fall | Participant 3 |
|  |  | […] And now, it's the complete opposite. I find myself feeling like an old man… I’m not exactly disappointed, but still, I'm suddenly confronted with my age, something I hadn't been preoccupied with before. | Participant 10 |
|  |  | We have suddenly become dependent on our children while we were independent. 100 percent independent.  *Quotation also relates to subcode 2: guilt (family and other informal caregivers)* | Participant 10 |
|  |  | I don’t want to go back home because of the fear for falling | Participant 11 |
|  |  | I also have … and that's just how I am. I want to do it by myself. Then I moved a bit. But I don't do much else yet, I don't dare that yet. | Participant 17 |
|  |  | No, just the fear of falling. If it happens once, at some point, that will be the end of me. | Participant 16 |
| **2. Guilt** | Guilt is an important concept among older hospitalized patients. By ending up in hospital, patients feel that they are encumbering the already busy lives of their children, who now have to take care of one and sometimes two extra family members. As such, patients are not only burdening their children, but also falling short in caring for their frail partner. Feeling loss of control is also present. The ability to help others and do your part – by watering someone’s flowers while they are on holiday, chatting with a lonely neighbor or helping children - are things that contribute to happiness, satisfaction, and well-being. The inability to reciprocate, due to ill health - undermines the morale of older patients. | | |
|  | Subcode 1: **Family and other informal caregivers** | Yes... that you have a good time together... that's quality of life.. | Participant 6 |
|  |  | […] Perhaps getting to the essence of what life is and what you have and what is important. The most important thing is your family and your partner. | Participant 10 |
|  |  | At least we had it good together. And that is very important. | Participant 11 |
|  |  | That little girl X [his daughter] does a lot of the shopping...  Interviewer: Yes.  She also has a card of mine so she can do the shopping, not with her own money but with mine... because I don't want her to run into problems [...]  Interviewer: So, your daughter X is really your caregiver?  Yes, yes... she doesn't forget anything...  Interviewer: And is she also the contact person here in the hospital?  Yes, she is also the contact person in the hospital. | Participant 7 |
|  |  | Yes, if she can, she comes... but I shouldn't make a habit of bothering her every time... but if there's really something wrong... she says: you can always call me. | Participant 2 |
|  |  | I spoke with him earlier this week, and he said, "I feel so lonely, and then I thought, darn... for him it’s the worst. | Participant 6 |
|  |  | I really hope that I can join him in the recovery home.  Well, you know... if I move to that facility, I'll obviously have a different physiotherapist than here, but they mentioned... it's only three times a week... and they found that a bit unfortunate... but on the other hand, should I be separated from my husband for that? Me here and him there? That would be tough on him...  Interviewer: And you feel it's more important to be with him?  Yes, absolutely. | Participant 6 |
|  |  | No... it's not that bad... I don’t feel sad quickly... I am a bit afraid... I hope everything, yes... [silence] it is what it is.. I have to look forward... as long as I can be with my husband... when I hear him say, 'I am so lonely,' then I feel so powerless. | Participant 6 |
|  |  | [...] I don't discuss it with the nursing staff... he knows that I find it difficult... that I find it the worst for him... he feels so alone, I have known that quite quickly. | Participant 6 |
|  |  | Yes, and eventually, it becomes too much, and then you have to give up... and the children can't do everything either... they can't sit with you at night... but still... they all have their families and the grandchildren are there... they have their own lives. | Participant 3 |
|  |  | I told that I have no idea what to expect... I have no experience with it... but I have chosen.... to have the shortest possible route for the people I'm visiting... because otherwise, they have to go to X, which is very far for them […] that's why I choose that option. | Participant 8 |
|  |  | Absolutely... without someone like that [an informal caregiver], you have a lot more worries and stress... when you're in such a condition and you have absolutely no one, how does that work? Who takes care of your laundry, who takes care of the mail?  Interviewer: But you're not worried about that at the moment?  No, because I know she takes care of all that... I'll take a taxi later, then the nurse comes and says, "Sir, you have to pay this much...I say... you don't think I'm walking around here with a lot of money or have a credit card on me? I have one, but it's in a safe, not in my pocket... you don't get assistance with that. I say, well, how much does it cost then? Yes, this much... so I called my neighbor again, and she had to bring money again yesterday... but if you don't have those people, how does that work then? | Participant 8 |
|  |  | My only concern was, ‘what have I done? What have I messed up?’ I used to be someone full of life. | Participant 10 |
|  |  | She [sick partner] suddenly had to do this and that and so on. It affected me so much.. It feels like I took something away from her by falling… a sense of security | Participant 10 |
|  |  | […] Yes, yes, yes. He'll be coming again tomorrow because he (participant's child) has his own things to attend to. I'm not the type to say, "You haven't been here for a day, now you must come, because he also has his own responsibilities. | Participant 11 |
|  |  | I always think about them [family] . I feel like they have to carve out time for me, even though I have plenty of time. I think, why should they be at my disposal? | Participant 12 |
|  |  | They [hospital] offered me another place to recover, but I don’t want to go there. I have to think about my children. Going there means a longer journey for them. | Participant 12 |
|  |  | You can be here, but you never know. Being here or, in my case, there's still work to be done. But then they have to take some measures at home again. And I have a few children, but they also have a family to attend to. | Participant 17 |
|  |  | Well, it's not that I have to worry about that. We'll manage that again. But I actually want to spare my family... how should I say it. I think if I had to rehabilitate, and I go to rehabilitation here [close to home].. | Participant 17 |
|  |  | Well, it's more for them than for myself. I don't want to burden them at home. If I still had a husband or children living at home, then I would have had some help. | Participant 17 |
|  |  | […] I tried that, and then I noticed that things weren't progressing as quickly as I thought. I still experience pain, and especially that sense of breathlessness lingers, making me realize that my recovery isn't as speedy. It means I can't go home yet. And I shouldn't rush it at home either, for then I will burden my children. | Participant 20 |
|  |  | I would like to be discharged to a nursing/rehabilitation home nearby. I prefer to go there because it's close by, also easier for my wife. Right now, I'm half an hour away from home, and I would like to be closer to home." | Participant 20 |
|  |  | I discuss it with my daughter when she comes. Because, of course, she can't always be with me. And I understand that very well. And, um, the nurses... I actually get along with everyone." | Participant 14 |
|  |  | I would have wanted to go home because he [partner] wants me to come home. | Participant 21 |
|  |  | Yes, that's nice to know, that he [sick partner] is well taken care of. They [the children] go to him every day, and I already arranged the meals, so that’s settled. And for the rest, well, I think he's doing quite well. | Participant 21 |
|  |  | Despite my eagerness to return home, I'm mindful that I shouldn't overwhelm my husband. | Participant 21 |
|  |  | […] It's a familiar spot, the recovery home. Why do I want to rehabilitate there? Well, because I want to be by his side. My husband has visual challenges, but he can move around carefully with his walker. It's a place he can easily maneuver his walker, it is suitable for him, and therefore for me as well. | Participant 19 |
|  |  | I want to go home but, in a decent way, that they're [children] not burdened with a parent they have to take care for all day..." | Participant 5 |
|  |  | […] Yes, I thought so... that I would, uh... but I'm not sure about that either. That's the thing. I've lost all the things I need to be sure about, and it’s difficult..." | Participant 1 |
|  |  | I've had a good life. | Participant 3 |
|  |  | I want to continue if things stay as they are now, and otherwise, I don't mind if it's over. | Participant 3 |
|  |  | ... I want to be resuscitated, but not if I'm, uh, mentally ill... then, I don't want to go on. But still, I want to stay with it... | Participant 4 |
|  |  | Well, at that age, you don't have goals anymore. No, because I've lived my life like.... I always say, I am unwishfully happy… | Participant 12 |
|  |  | [..] Oh, you just have to endure it [the situation you are in]. Complaining doesn't help. Fortunately, I'm still in a good mental health. | Participant 14 |
|  |  | In the long run, you need to ask for help... However, the children cannot do everything [...] they can’t sit with at night. They have their own families and grandchildren. My children all have their own lives to get on with. | Participant 3 |
|  |  | If she [daughter] can she’ll come... but I shouldn’t make a habit of bothering her… but if there’s something serious than I can call her | Participant 6 |
|  | Subcode 2: **Feelings towards others** | […] My husband’s so sweet... and I could still do it... [silence] my husband was such a good soul... and he... you don't let him down, do you? You don't let a stranger do that? I could still do it [help]. | Participant 3 |
|  |  | […] Yeah... like I've been saying... I can bring people here a nice cup of coffee, those who are in wheelchairs... I put the coffee on the walker... it's a way to connect, you know... | Participant 3 |
|  |  | I said ‘It's winter, but I'll pick you up around coffee time. We'll stay at the garden, and if you've had enough, we'll bring you back home.’ [silence] Because It’s important, to care for others. | Participant 8 |
|  |  | […] [silence] I wouldn't know... [silence] no, I wouldn't know what I really need... well, [silence] I want people around me, yes, people around me. That's what I like the most. And that's not always possible, of course... at least not here." | Participant 1 |
|  |  | What I have seen there... for example, in X... when I visited an old singer there, I would come in, and then you sit at the table, everyone is sleeping... the television is on, and no one is watching, they don't talk to each other... I like to make contact, have conversations about the past, about the present... I would go crazy there, really.  Interviewer: "So, you miss the interaction with your fellow human beings?  I just want to stay among people... let me tell you, | Participant 8 |
|  |  | While our world becomes smaller as we grow older, we are still very happy. We have no worries and live in a nice community.  Interviewer: that sounds nice.  My husband is still very emotional about what happened. He has the tendency to cry when he sees me, so I tell him: ‘I’ll be back…’ yes, there comes a time that one of us will go, of course. | Participant 21 |
|  |  | Human contact is the most important thing there is. | Participant 19 |
|  |  | […] …And when I woke at night, I sat in the room, and started drawing, sketching again...  Interviewer: to pass the time?  Yes, to pass the time. And I still read the paper. I don’t want to miss what’s happening in daily life.  Interviewer: do you find it important to stay informed?  Yes, of course. If not, I would have not known so much as I do now. It’s your duty, as a citizen, to keep yourself updated, even if you’re old. | Participant 7 |
|  |  | Yeah, but honestly, while it's fantastic that all these amenities are here. However, I don't really take advantage of them. I mainly spend my time watching TV because I like to stay updated. | Participant 3 |
|  |  | I like to stay updated. For example, It was on the news this morning that Boris Johnson visited India. Knowing what’s happening keeps mee sane and gives me a sense of self. When my husband or someone drops by, I often ask them to switch on BBC One when they leave. | Participant 19 |
|  |  | I would like to be discharged to a rehabilitation home nearby. This is where I would like to go because then it’s nearby [...] that is easier for my wife. Now it is a half hour away, I would like to stay closer at home. | Participant 20 |
| **Theme 2 – Patients in the passenger seat** | | |  |
| **1. Independence** | Independence is about older patients’ ability to perform physical activities unaided. Remaining independent is of paramount importance and therefore often an end in itself. Because the need to maintain or recover an optimal level of physical functioning is so high, older patients view recovery as something practical; accepting both physical help and material help in order to regain functional ability as quickly as possible. Although patients are severely limited in their independence during early hospitalization, they are surprisingly optimistic about their recovery. Older patients are prepared to see the glass half full rather than half empty. This goes for both acute illnesses, which are related to the fall, and chronic issues. | | |
|  | Subcode 1: **Goalsetting to remain independent** | But, I try as much as I can and I do. Because this morning in bed, while you guys were still busy, I immediately.. you know [moves] with the arms and so... and so and so.... and a little bit with the shoulders and so and so. | Participant 2 |
|  |  | Well just like everyone else, walking again and so I can just do that again.... if I can do some things again.... | Participant 6 |
|  |  | Once a week I have help.... two hours... well I mean... on Mondays I do some myself and on Fridays she [the help] does it, or on weekends.... and that's okay... and I'm fine with that and cooking and all that and the shopping I do.... no.... I do the shopping at the market... but I'm not allowed to go to the grocery store, with one of those facemasks.... [laughs]  interviewer: [laughs]... Yes.... you indicate that before admission you were very independent and your goal is to walk relatively long distances again after admission....  yes  interviewer: Have you made that known to anyone? or are you keeping that to yourself?  Yes, well, that goes without saying I think....  interviewer: Yes...  You don’t want to stay at the same situation | Participant 6 |
|  |  | No, I think so... want to walk... Yes, if I can.... if I can keep doing my grocery shopping and so on, I think, well... go ahead... | Participant 6 |
|  |  | Interviewer: And what does that mean to you? Because you keep losing something now.... At first you could walk, now that's also becoming less. The memory is deteriorating.  I have a walker that I walk with...  Interviewer: What is important to you in life?  Walking  Interviewer: Walking..  Otherwise, you get stiff | Participant 9 |
|  |  | I won't be alone anymore... because I was still cooking by myself, frying, bathing, cleaning.... | Participant 3 |
|  |  | As long as I don't get clumsier and forget things..... | Participant 3 |
|  |  | But then I got a crooked mouth and then I didn't blow anymore.... I was very sorry about that....  Interviewer: Yes.... those are things you like and then have to let go.....  Yes, that is a loss.... my husband who never whistles.... and those guys said: dad, so...... and he said -I can't do it, cut it out-.... and then I was judging him... yes but try it now... yes and for punishment I suddenly couldn't do it through my mouth either.... I think I shouldn't have tormented that man then [laughs].  interviewer: Yes [laughs]  It was the same when I was in bed.... then I lay watching... yes that eye didn't close anymore either.... then I could hear it... then he said... -are you going to sleep or not?.. Yes you are still watching-.... then I said no, that's that eye.... [laughs] [silence] yes I say, I've had a nice life though | Participant 3 |
|  |  | That I get better soon. As far as.... That I will be somewhat independent.... | Participant 3 |
|  |  | I just surrender to that.... they often say, you will live to be a hundred years, but now that I am X years old then I think.... yes guys you should listen... people of X years are much worse..... so, I experience it, but I also accept it....  *(also related to subcode 1.3 and 1.4)* | Participant 8 |
|  |  | interviewer: Are you helped to get out of bed?  If eh ... if I asked then yes... but I always want to do a lot by myself.... because then you are more involved in the recovery and the progress will be faster… Well, this morning I had physical therapy [...] I know what time he [physical therapist] comes and then I try to sit on the edge [of the bed] before he arrives | Participant 4 |
|  |  | ... I just want to be resuscitated, but not if I'm eh crazy.... because then I don't have to live anymore. I still want to stay clear-headed... | Participant 4 |
|  |  | And neither did the other one. But enough is enough I always say. No, come on. Once we've had it. We can't all live to be a hundred. Look, I try too. But, no, I'll go anywhere I want to be. | Participant 12 |
|  |  | Interviewer: Now you are somewhat independent and can walk by yourself, do you like that?  Yes, I think that's ideal. Sure. And I live towards that. Sure. Because the sooner, sooner, I can walk on my own, and then they send me home. Look, there's something behind me, with them, look. | Participant 12 |
|  |  | Yes, I still actually went to the toilet, under supervision, though. But yes, they do say sometimes.. -yes, you are already ninety-one but um we don't experience that much-, that's why I’m kind of proud of. | Participant 18 |
|  |  | Interviewer: And can you also indicate what you like yourself? In what order or what you like to achieve?  No, well. What I would like to achieve is that I will go home later.  Interviewer: Yes  And not fall down me. And then I have to be able to climb stairs. | Participant 15 |
|  |  | Now you can walk a little better, stand a little better. Then I can get in the shower and just let it run. And then I do get clean at some point. Anyway, I can stand again. | Participant 15 |
|  |  | Yes, that is in my nature, wanting to be independent and figure it out on my own. | Participant 20 |
|  |  | I just need to actually learn to walk again.  Interviewer: Yes because I also see a walker standing there?  Yes, and so they try to make me walk behind that. I do have to make sure it's unlocked. First it has to stay locked.  Interviewer: Yes  And then comes the person who is going to walk with me. And then we put it off the lock. And then um, this leg is good. And then I just try to walk.  Interviewer: And how is that going? How do you find yourself doing?  It's coming. It's coming. | Participant 14 |
|  |  | Then you get a bit of an ego again because actually you want to do it yourself. | Participant 13 |
|  |  | Oh yes, you still practice about it sometimes. But, then I also think yes, it happened that way. You're rehabilitate and you're trying to get back to your old self as soon as possible. | Participant 13 |
|  |  | Anyway, I can stand again. I can walk with a walker. But it does hurt. | Participant 16 |
|  |  | ‘’You said that you were very independent before admission and your goal is to walk relatively long distances again after admission....’’ –interviewer.  ‘’Yes’’  ‘’Did you tell anyone about your goal? Or are you keeping it to yourself?’ -interviewer  ‘’Yes, well… I think it’s obvious. ’You don’t want to stay in this situation’’ | Participant 6 |
|  | Subcode 2: R**esponsibility towards regaining independence** | I have to do exercises myself, of course.... | Participant 2 |
|  |  | But try as much as I can and I do. Because this morning in bed, while you were still busy, I right [moves] you know with the arms and so... and so and so.... and a little bit with the shoulders and so and so [shows exercises]. | Participant 2 |
|  |  | Interviewer: yes..... um, we have quite a tight regime here.... you wake up... wash, dress, have breakfast, exercise with the physio..... what do you think of that daily regularity?  Well, it's easy.... they said that from here too... there is only a physio three times a week and they actually didn't like that.... but you can do your own exercise in between?  Interviewer: You can certainly exercise in between... do you think that is your own responsibility to get better?  Yes I do... you have to get better yourself and you have to do something for that... | Participant 6 |
|  |  | If I say, don't do it like that, then she says wait but I will do it... but she leaves a lot to do for me because I want to do it by myself... and I have to because otherwise you won't get there... you also have to take the wheel by yourself. | Participant 3 |
|  |  | No, I'm happy, sometimes it's hard, but you have to do it anyway, even if you don't feel like it (I: yes) but you do it anyway (I: yes). Well, the benefit can now be seen. | Participant 18 |
|  |  | Ideally, I want to go home. But then I have to try very hard, because I have to be able to climb the stairs, and that is not realistic at the moment. | Participant 15 |
|  |  | ..physio. So yes, they did the first steps. And then you still have to do it yourself. | Participant 15 |
|  |  | Yes. Well, they can't do anything about it here. It has to heal itself. | Participant 16 |
|  |  | For example, I am doing more and more by myself. For example, after two days I got out of bed on my own and since yesterday I have been going to the toilet on my own. | Participant 20 |
|  |  | But I do know several exercises, so I'll just do those. | Participant 21 |
|  |  | Yes, I find it does little to me in that sense because I think it's my own fault. I'm not like, why is this happening to me? | Participant 20 |
|  | Subcode 1.3: **Accepting help to regain independence** | Yes, of course... basically I would have liked to do it all by myself, that I… I do know that's not possible so then it's nice to have help.... | Participant 1 |
|  |  | [silence] Now if I have to I'll stand up for myself.... Well otherwise this couldn't be so I had to. | Participant 4 |
|  |  | Ah but they usually let...What you can do yourself you should do yourself. | Participant 18 |
|  |  | Interviewer: Yes indeed [laughs]. And further in terms of care, I think at first you just did that completely by yourself. Washing yourself, refreshing yourself....  But I can't do that anymore.  Interviewer: Yes  No, you have to help me with that.  Interviewer: You do get help with that?  Yes, I did it on my own this morning but it's still a bit of a struggle though. | Participant 15 |
|  |  | I submit to that. I do notice that this time it’s different, and especially because of my shortness of breath. | Participant 20 |
|  |  | Yes, I was always walking with my walker. Because I have a fixed back. And that didn't happen here.  *(also related to subcode 1.4)* | Participant 14 |
|  |  | Interviewer: And how is that for you?    Well, I have to surrender to that.    Interviewer: And will that be possible for you?    Yes, it will have to. | Participant 21 |
|  | Subcode 1.4: **Attitude towards recovery** | Um and I have no barrier to get outside. And if it becomes with a walking stick or with a walker, I care very little.... | Participant 5 |
|  |  | My recovery... no because even if I could walk, I would still try to take an artificial leg. | Participant 7 |
|  |  | Then it would have been um, more difficult. Now I can still pick up my cup of tea and my sandwich and so on.  interviewer: Yes... do you like the fact that you can still do things yourself?  Yes, I like that very much,.... look [demonstrates]. I can do all that myself. Only cutting something is difficult. But they helped me, yes.... | Participant 2 |
|  |  | Well just like everyone else, walking again and so that I can just do that again.... if I can do some things again.... | Participant 6 |
|  |  | Last year we were talking about… such an apartment.... because it's huge anyway... I have three floors.... but now with that stair elevator.... so wonderful... then I can stay here... yeah I mean... | Participant 6 |
|  |  | I have a walker that I walk with...  Interviewer: What is important to you in life?  Walking  interviewer: Walking  Otherwise, you get stiff | Participant 9 |
|  |  | Yes, I hope that walking is going well again... that.... of course, that's always nice and then of course you hope that's going to be okay again [patient walks with great difficulty using a walker] | Participant 1 |
|  |  | interviewer: If you had been sitting here in your pajamas....  No, absolutely not, no. I would not have wanted that  interviewer: You wouldn't have wanted that... why not?  I don't know... yes I do know, then you have the idea that you are sick. Now I have the idea that I am healthy... maybe I am not at all but I feel healthy.... yes. | Participant 1 |
|  |  | Yes.... trial and error though... life isn’t easy... everything... there are setbacks too... but yes [silence] you have to be able to cope and get up... | Participant 3 |
|  |  | Well, I had time for myself.... if it takes me half an hour, that doesn't interest me then... the idea of I can do all that by myself.... that is important... also for my self-esteem... | Participant 8 |
|  |  | No, because that makes no sense.. then you are only dealing with yourself… | Participant 8 |
|  |  | ‬Well, the expectation is that I may not have any expectation for the first six months.  Interviewer: That's what was said?  That's what has been said.  Interviewer: What did that mean to you?  I put it aside because I think, that's how it is. Fortunately, I don't live in a flat. I can walk into the garden and we have a lot of freedom. | Participant 10 |
|  |  | … I don't have a car anymore. My car is disapproved. Or at least disapproved… I could have had it repaired but that wasn't worth it... I thought, before I have to do it again then I'll be another year down the road and by then it'll be over and I won't even have had fun with that money, so out with it. So now I am dependent on the ‘Beltaxi, or ‘het busje komt zo’ [taxicab]. I am already a member of that. Our kids took care of that years ago. Then you have to call two days in advance and then half an hour in advance and half an hour ... so well, so that becomes a call cab. But I think about all that. I think, I can judge that now but I'll see.  Interviewer: So that doesn't hinder you.  It's not important. | Participant 10 |
|  |  | I started experimenting myself. I'm a technical guy, let me put that first. It was an issue for a while, right and left. So, I was already doing a very little bit of ... how do I get out of the hallway? I'll be fine. | Participant 10 |
|  |  | Well, it hurts because yes ... but other than that ... you are, except for the pain, healthy | Participant 4 |
|  |  | Um, no... that's eh, because there was no other way I had to have surgery, which was the only option and the only positive thing, that I could still have surgery.... | Participant 4 |
|  |  | Because then you have to try to keep walking anyway, with a walker, for example.... then you walk across the yard again anyway.... you've always done that, so you always keep doing that automatically and then you keep monitoring it.... | Participant 4 |
|  |  | I can say that's nothing for me, but a certain age, and I'm 87, you have to start using that anyway. | Participant 12 |
|  |  | Interviewer: Did you have to be sober for a long time, for example?  No, but that's not a problem for me. So, I don't think about that you know.  Interviewer: And are there any things like complications and so on? Have things like that been discussed with you? Of, what to expect after the surgery?  I think everything has been discussed. | Participant 12 |
|  |  | There is nothing wrong with me. | Participant 12 |
|  |  | You don’t have no longer goals at that age. No, because I lived my life as a... . I always say, I'm grateful and happy. First, my children are healthy. That is the most important thing with me. My grandchildren are healthy. And I'm not missing anything. | Participant 12 |
|  |  | No. What comes that comes. No, I am now reassured, I have had the surgery. So now we're just going to work on recovery. And if I.., from here I'm going to a revalidation Centre. | Participant 12 |
|  |  | Yes, it's not that bad. It's always like that at the beginning it's still too stiff and then a little further on, halfway through, it's better. And then all the way back again. | Participant 18 |
|  |  | Interviewer: But how is the recovery going?  Well, it is progressing.  Interviewer: Are you still suffering from you hip, pain?  Pain yes, God, it must be yes. That woman [pointing to other patient in the room] has been in pain for six weeks. So, I just wait and see. But I try my best. | Participant 15 |
|  |  | Yes physio. So yes, they did the first steps. And then you still have to do it yourself. | Participant 15 |
|  |  | Well, I can't walk very well. And, but yes, it's not Monday yet. It can still get better. | Participant 17 |
|  |  | I had surgery on Tuesday and I walked a little bit since then. But yes, that's not much really. | Participant 17 |
|  |  | I live also still at home by myself and I can do a lot myself. Before corona, of course, it was even better. But with corona ... I did take a dip for a while but I was just like, I'm saving myself again. | Participant 17 |
|  |  | Yes, it all went well. I haven't had a fever yet; my blood pressure was good. | Participant 17 |
|  |  | I did that and then I noticed that things were not going so fast. I still have pain and especially that tightness of the chest remains present so I notice that I am not recovering that fast yet. That I can't go home yet. And I shouldn't do that at home either. | Participant 20 |
|  |  | For example, I am doing more and more by myself. For example, after two days I got out of bed on my own and now since yesterday I am going to the toilet on my own. | Participant 20 |
|  |  | No, I don't have to. I try to lie down a little bit myself and try to go back to sleep. I also don't get out of bed to pee. I pee in the bottle. But yes, then you are awake again for a while. I have not had a good night sleep yet. | Participant 20 |
|  |  | Interviewer: But the sport which you love so much? Are you going to stop doing that?  Well, not stop completely. Who knows, I might soon be able to do more again than I think now.  Interviewer: Yes  I can also whistle at 10 to 12 year olds [soccer coach]. Then I don't have to run as much. And of course I can go to the gym.    Interviewer: Yes  Not for bigger muscles, but just to keep moving. Yes, I will still keep doing that, going to the gym. | Participant 20 |
|  |  | Interviewer: And then, when you look back on those past days: how did that go?    Well, a lot of pain, right?    Interviewer: Yes.    Yes. But other than that, it's going pretty well. We're going to deal with it. | Participant 21 |
|  |  | So. And that was it, wasn't it? Yes, we do keep our spirits up, because I can grumble: oh, difficult and all that. But that doesn't help either. So. And you're well taken care of. | Participant 21 |
|  |  | And exercises I had to do on my bed.    Interviewer: Yes.    And that's what I'm going to do, too, you know, that all of a sudden it's a lot better on Monday. | Participant 21 |
|  |  | Yes, right? You can't change it anyway. And I just stay positive. | Participant 21 |
|  |  | Well, I... at the moment I can't do anything. It's um, you want it so badly.. | Participant 13 |
|  |  | If it were up to me it would all take way too long. | Participant 13 |
|  |  | Oh yes, you still practice about it sometimes. But, then I also think yes, it happened that way. You're recovering and you're trying to get back to your old self as soon as possible. | Participant 13 |
|  |  | I wouldn't say, I think, I don't say bad luck!  Interviewer: Yes, that's how you see it? Yes.  Such as life.  Interviewer: Yes, well we've been sitting for over an hour.  Yes, it was a nice talk. And my family will tell you, "Be careful, she likes to talk!  Interviewer: Well, that's a good trait right? It’s better to vent than bottle it up.  Well, my characteristic has taken me very far. That's why I can teach about it in college. I do a lot of games, the students very often ask me, "Misses [name], can you give us a game?". Because I arrange games depending on what I think they need. And suppose, and I call that 'stretch,' and a 'stretch,' you know to stretch. And a stretch is something, it should take you out of your comfort zone. And I'm sitting here, I have a notebook and a pen. Just so, I see something and I think, "Oh, that would be a nice stretch. | Participant 19 |
|  |  | Eh, there are no barriers to getting outside the house. If it means I need to use curettages or a walker… well, that interests me very little. | Participant 5 |
|  |  | Yes, it is not too bad. It is always like that in the morning. It’s [the hip] is stiff, but halfway through the day it gets better and eventually back to the way it was [before injury]. | Participant 18 |
|  |  | But, the sport that you love so much… are you going to stop doing that?’’ – Interviewer  ‘’Well, not completely stop. Who knows, soon I might be able to do more than I think right now. […] and I can also revery  The 10-to-12-year-olds, than I won’t have to run as much. And I can visit the gym, of course. […] Not to tone my muscles, but to remain active. Yes, I’ll still keep doing that, visiting the gym. | Participant 20 |
| **2. Personal Autonomy** | Personal autonomy is about acting on one’s own behalf and choosing what care one wants and needs, and where and when that care should take place. When patients have personal autonomy, they are in control. They have a feeling of self-direction. Personal autonomy can be influenced by an alien hospital environment, acute illness, but also by the trust older patients have in the expertise of healthcare professionals and/or family. Authority – especially that of doctors - can also affect personal autonomy. Assertiveness seems to be an important feature to assert personal autonomy. When a patient informs a healthcare professional of their wishes and needs, the caregiver will go along with this. When older patients suspend autonomy, care is quicky taken over by healthcare professionals, leaving older patients feeling loss of control over their situation and health. | | |
|  | Subcode 1: **self-direction** | No, but I um... They are busy with um... Yesterday I was called by um about that, by a firm, um which they've been in touch with.... | Participant 5 |
|  |  | This week I'm going to spend a day with my daughter to put things on paper for the funeral.... that she will know what to do when the times comes. It just has to be well organized. | Participant 7 |
|  |  | I say to the doctor, can you write it down because otherwise I'll don’t remember it in no time.... that's easier for me... | Participant 9 |
|  |  | Yes, for example... I'm not in pain very often and I don't need paracetamol very often but I think they would give it if I ask for it. I don't know if that's still be the case, but when they give my medicine they offer me always paracetamol but I didn't really need it. Sometimes I would take it and sometimes I wouldn't. [silence] | Participant 1 |
|  |  | What matters is that I have to walk,... but before I was independent.... and when I wash myself I do the suggestion that I don’t need help but they have to stay with me.. just in case if I fall. | Participant 3 |
|  |  | If I say I don't be like that, she says wait but I'll do it for you. But she leaves a lot for me to do by myself because I have to... because otherwise you won't get there.... you have to take control.  *(also related to subcode 2.3)* | Participant 3 |
|  |  | Yes Saturday they did a scan.... `  interviewer: Did they say why they wanted to do that scan?  Well, I wanted that myself.... if something is torn, they might be able to find it with a scan | Participant 8 |
|  |  | No, but I say guys I can do all that myself....  interviewer: You said that by yourself?  Yes  interviewer: And that was considered? It was not taken over?  Do you know how to put on the undershirt? Just let me go for a moment and then I did that and then I said now you have to help with the shirt on the back, to put it on neatly. | Participant 8 |
|  |  | No, I just told that then again... I didn't mind at all. But what I can do it myself that I want to do it myself.... and now they have to help me?  interviewer: Do you think that's important too?  Yes.... the most important thing is to keep doing as much as possible yourself....  Why is that important?  That independence... due to the X [disease] I was so tired.... so tired.... and back then I would peel two potatoes and then I had to sit on a stool because I couldn't stand at the kitchen counter.... last week I didn't have to... so I mean you feel you've gotten a lot better.... | Participant 8 |
|  |  | Well, I had all the time....al if it takes me half an hour, which doesn't interest me then... the idea of I can do all that by myself.... that is important... also for my self-esteem... | Participant 8 |
|  |  | Because you are alone, you have to take care of yourself and arrange things yourself. By already writing these things down, it brings peace of mind. Because you have to arrange everything. | Participant 8 |
|  |  | I think, should I then sit down and shower and all that by myself ... I think, yes I would like that quite a bit. Put me in that chair and I'll take a shower and you guys will come later. I had that idea. But of course, there is a shower in the shared room. | Participant 10 |
|  |  | That was a boost so I immediately called the whole family. | Participant 10 |
|  |  | Interviewer: You say there has already been a conversation with you about a rehabilitation center. You may be going to a nursing home. Has this information been discussed with you or with a family contact, such as your brother?  No, it was discussed with me.  Interviewer: So, it was discussed with you?  Yes.  Interviewer: Did they explain that in easy language, did you understand everything that was told?  Yes. I have a tremendous understanding, of course. No way, it all went fine. | Participant 11 |
|  |  | interviewer: Do you receive assistance with getting out of bed?  If ah... if I ask yes... but I always want to do a lot myself.... because then again you are involved sooner and then the recovery progresses faster.... Well this morning I had physical therapy.... | Participant 4 |
|  |  | I like it when it's asked and I can do as much as possible myself and eh that they stay with me and they do give me the opportunity to do that.... | Participant 4 |
|  |  | I couldn't put on my sports pants myself at first. That I have to bend down to put them on, that's not possible because of the pain, because of my ribs.  Interviewer: Yes  I am that person that I still want to do it myself, I will try something until I succeed. I get my sports pants over my ankle by doing this with my other foot, with my big toe. Then it succeeds.  Interviewer: So, then you still manage to do it yourself?  Yes. But other than that, I still need help. That is why we have already talked about a rehabilitation center. | Participant 20 |
|  |  | Interviewer: And your wife, is she involved in the plans about discharge?  She is involved yes; I call her twice a day and she visit every afternoon. I can talk easily with her and we haven't had any words yet. Yes, she is involved. | Participant 20 |
|  |  | But yes, what I can do by myself, I will do myself. | Participant 21 |
|  |  | Interviewer: Do you then tell your daughters how it's going, or do they sometimes have a conversation with the doctor?  Well not especially  Interviewer: No  I usually tell them myself | Participant 13 |
|  | Subcode 2: **Power ratio between patient and professional** | … and my daughter was sitting next to me on the couch poking me, and she says afterwards, you can't talk to the doctor like that.... | Participant 7 |
|  |  | … a very good doctor who said, "madam, you can't go home yet...I was sorry about that... "because you are very sick...but I will see you every time when I come to the ward... Well... I liked that man... because he was the director... | Participant 2 |
|  |  | interviewer: okay... let's see.... yes, you said you are not very involved in care.... you are not seen as a conversation partner.... [patients interrupt]  no... not a conversation partner.... actually just.. [silence] ..it gets done.... and I find... just hear me... that's maybe also because I know a little bit how it is.... they think well... if they think this or that or say that it will be this or that.... That makes a difference... they said later... you must have been hospitalized. | Participant 6 |
|  |  | Well... now I wash myself again.... before it was not allowed | Participant 3 |
|  |  | interviewer: And is everything meeting your expectations.... ?  Yes.... [silence] no... you just accept everything... | Participant 3 |
|  |  | To me, it's just a person... the white uniform means nothing to me... I'm sometimes afraid... that there are nurses working here for the uniform, not for the purpose they stand for | Participant 8 |
|  |  | No, because I had received that task. As soon as I have something, I need to call ‬ | Participant 10 |
|  |  | Well, I have little to want for because my legs are paralyzed, so I have little to wish for  *(also related to subcode 3.1 and 3.4)* | Participant 11 |
|  |  | I assume that those who, who help you that those are skilled  Interviewer: Yes, yes  [clears throat], because um it's a hospital then um, a resignation of trust, yes | Participant 18 |
|  | Subcode 3: **Patient assertiveness** | Afterwards, things improved because I started asking for more myself. | Participant 5 |
|  |  | I say no, because I can stand and I can take a shower and I'll be fine and I'm doing that here. there are nurses standing by.... | Participant 7 |
|  |  | Yes, I do know that.... but if I really had things I would say it too, but it's going well the way it's going.... | Participant 6 |
|  |  | Interviewer: Did they ask what you can do yourself and what you cannot do yourself?  Yes they do.... I had to tell all that  interviewer: Did they ask about that?  No, I told.... | Participant 9 |
|  |  | Yes Saturday they did a scan.... `  interviewer: did they say why they wanted to do that scan?  well, I wanted to.... if something is torn, they might be able to find it with a scan | Participant 8 |
|  |  | No, but I say ‘guys I can do all that myself.... ‘  interviewer: You indicated that yourself?  Yes, I indicated that myself....  interviewer: and that was considered? It was not taken over?  Do you know how to put on the undershirt? Just let me go for a moment and then I did that and then I said now you have to help with the back. | Participant 8 |
|  |  | With pain you had to indicate what grade you were at, from one to ten.... what rating do you give the pain, they say... I said an eight and not lower.... then if they can do something with that then .. they said ‘If you have pain, I can’t feel your pain.. you have to tell me what you need.’ Then I just have to listen what to do and do everything very gently and not too wildly.. then you have as little pain as possible.  interviewer: Was there good listening to that?  Sometimes. Yes, but you have to bite through it, because otherwise it gets all stiff.... then I think where do you get that wisdom? Every person feels his own body best.... and how far he can go...  *(also related to subcode 4.4)* | Participant 8 |
|  |  | Are you helped to get out of bed?  If uhm... if I ask yes... but I always want to do a lot myself.... Because then you become involved more quickly, and the recovery is faster..... well, this morning I had physical therapy.... | Participant 4 |
|  |  | It happened during the first day. Then I say, doesn't that get dried off? No, she says, that dries itself. I say, you're a good one. I say, when you're washed you have to dry yourself. 'No that will dry itself in a minute'. I think, well, nothing for me. That's old school. I'm 87! | Participant 12 |
|  |  | Look... The thing is, if I say, I don't want it. Then it doesn't happen. Let's be honest. | Participant 15 |
|  |  | But I do have the feeling that doctors mainly focus on the recovery of the fracture and not on the rest, for example, my lungs. I had to ask myself if they wanted to check my lungs and hemoglobin. | Participant 20 |
|  |  | Well, they're trying to take over. I say, let me. Here you have a shower and a toilet for the patients. | Participant 14 |
|  |  | Yes, and when was it? Yesterday? And I say well, I told them, 'I know you guys are busy, so I grabbed one myself.' I tried to help a little bit.  Interviewer: Yes, and what did they think of that?  They told me... And then I said, 'But my back is still itchy.' Then she said, 'Okay, then let me take a look'. And she said, 'You don't have to use your hand'. And she said, she says, 'Use one of those wet gloves'. And she said, 'Okay, come on. I'll help you. Come on!' And she washed my side and my back.  Interviewer: Yes.  Everything is discussable. | Participant 19 |
|  |  | Interviewer: And are your complaints of pain taken seriously?  [silence] pff some may understand and some may not.... there's one of them, I think it's a professional woman, though.... sir we take both your legs and we throw them overboard like this.... I say we can't... You have to go through it because eh.... I won't take it... because I have the best feeling about how far I can go. | Participant 8 |
|  |  | When I said “don’t do it like that,” she said: “wait, I’ll do it.” But she left a lot for me [during ADL], because I wanted to do it myself… Otherwise, I won’t get there. You need to take control and steer the care yourself. | Participant 3 |
|  |  | The chaotic situation in the emergency room and the lack of information left me feeling incapacitated. As I was the victim, they [healthcare providers] merely communicated with the lady who accompanied me to the emergency room. | Participant 5 |
|  |  | I mostly start the conversation. Within an hour of arrival, I usually know who has a pet, who’s in a choir… I always look for common interests. […] I want to meet the person behind the uniform. To me, HPs are just people | participant 8 |
|  | Subcode 4: **Trust in professionals** | Interviewer: would you have liked it if it had been done?  Oh well, why? you know, don't you? [laughs] | Participant 6 |
|  |  | So... I assume, if a doctor approves it... who will know what's going on then I assume that's good for me. | Participant 8 |
|  |  | They know what's going on | Participant 8 |
|  |  | I'll take that into account because that is the professional and he has examined me and he knows how I can stand on it [hip]. | Participant 8 |
|  |  | Yes... [silence] they know more about it than I do.... | Participant 4 |
|  |  | Yeah, well, I usually ask... How it's going or something. I just wait and see. .. 'You need surgery on your hip,'… I say, well, I would have thought so, say. Because it can't heal like that, from the outside. Or in x-rays, you can do that too, if you can. That would be all right. No, it wouldn't. I trust them. | Participant 12 |
|  |  | I assume that those who, who help you that those are educated | Participant 18 |
|  |  | I assume that those who, who help you that those are skilled  Interviewer: Yes, yes  [clears throat] because um it's a hospital then um, a resignation of trust, yes | Participant 18 |
|  |  | .. because we are not experts and we assume that they are the experts  Interviewer; yes, yes, yes)  So those who mastered the craft. | Participant 18 |
|  |  | Well.. if she says so, we'll manage. | Participant 17 |
|  | Subcode 5: **Patients suspending control to family** | And, um, so that's good... I didn't really have any problems with it...  Interviewer: You didn't have any problems with it...  Because she's very reliable.  Interviewer: Okay...  And, um, she's been coming to our house for more than thirty years, and, um... what I was mainly concerned about is that she would inform my wife or the children.  Interviewer: That was the most important thing for you? That the family was informed?  At that moment, yes | Participant 5 |
|  |  | Of course, it's, uh... It happens to you. And if I had to do it, you know, and I've had to deal with quite a, uh, a lot of things in my life... And, uh, from homeless people to, you know, I don't know what. Yeah, then it's nice if, uh, if you can't do it yourself, that someone is there who, uh...  Interviewer: Yes.  ...can guide in a conversation and, uh... | Participant 5 |
|  |  | Actually, very little. Because my wife told me | Participant 5 |
|  |  | No, not at all... it just had to happen... I had to go upstairs, and then... my partner arranged it | Participant 6 |
|  |  | Yes, my sister, she always goes with me, and she knows everything... see, if you call them, she can exactly say what was said.  Interviewer: So, was everything discussed with you or with your sister?  With my sister.  Interviewer: Don't you mind that?  No, I find it comforting. I can't remember it, and then I want to say it, but I don't remember it... | Participant 9 |
|  |  | Interviewer: Do you already know something about the discharge?  Discharge? No... I don't know anything.  Interviewer: Is it discussed with you, or is it may be discussed with your sister?  With my sister... she must know... I don't know... she will call because she's in charge...  Interviewer: Do you like that everything is discussed with her?  If they tell me, I'll forget it quickly. | Participant 9 |
|  |  | I have no idea. I would have to ask my daughter. I don't remember. | Participant 1 |
|  |  | Interviewer: What would happen to you after the admission? Has that been discussed with you?  No... they happened to have a spot, and I'll leave the rest to my children...  Interviewer: Is it the same when decisions are made here?  Yes, it's all discussed with the children...  Interviewer: Didn't you want to be involved in the decision-making yourself?  Well, [silence] but the children... the children do what's best for me... I used to do the same for them in the past. | Participant 3 |
|  |  | Interviewer: Didn't you have a desire to be involved in the decision-making yourself?  Yes, [silence] but the children... the children do what's best for me... I used to do the same for them in the past. | Participant 3 |
|  |  | No, there have been no family discussions... everything was done over the phone... my son and daughter-in-law do the talking...  Interviewer: Do you have confidence that they represent your interests during such a conversation?  Yes... yes, my son knows what I consider important. | Participant 3 |
|  |  | Interviewer: Would you share these kinds of feelings and things with the nurses, for example?  No, you don't share that with the staff. I also think that if I didn't have someone to rely on, the doctor would probably say, "put him in a nursing home and let him spend his final years there, and that's something I really don't want... because then I would lose all my contacts here on the street... and, that's not a life for me. I'm still far too active for that. | Participant 8 |
|  |  | At that time, the fortunate thing was that the daughter-in-law was visiting her father in [city]. She immediately took over the task. She's a nurse." | Participant 10 |
|  |  | At first, it was up to me because she wasn't there yet. But then, when she was there at some point, I was told that help was coming from outside the family. But how... I don't know. Those are questions that are gone now because you're asking, they are somewhere. But I was glad when my daughter-in-law came, and she arranged things again. Of course, she couldn't help medically, but I was happy that she was there. She kept me calm. | Participant 10 |
|  |  | Interviewer: Because he calls or addresses the nurse himself?  He asks the nurse himself... then he, for example, asks what happened, and yeah, he keeps an eye on that.  Interviewer: Is there a difference in what you find important in care and what your family finds important?  It's the same... we think very much the same. | Participant 4 |
|  |  | Yes, at least, they already have it... I think they already have it... Because she said that. And my daughter-in-law...  Interviewer: She, the nurse said that, or someone else?  No, someone else. But she says, we're going to work on that. But José, she already asked. Because it's easy for her too, as she works in that hospital. And then she comes to me in the afternoon, just for a quarter of an hour, you know. | Participant 12 |
|  |  | Interviewer: Yes, so you can easily rely on that if needed.  Yes, always. | Participant 12 |
|  |  | Interviewer: Yes. And was your wife also aware of the rehabilitation plans?  Well, she's a physiotherapist. She knows what she's talking about.  Interviewer: She knows what she's talking about.  She knows whether I can go home or not. And yes, we said, let's give it a try, and if I do my best, I can go home.  *(also related to subcode 4.2)* | Participant 15 |
|  |  | My daughter was really scared. My daughter comes to see me very faithfully and is very sweet to me.  Interviewer: Yes.  I was always sweet to her too. Because I, um... | Participant 14 |
|  |  | But otherwise, I really... I don't know. My daughter takes care of my underwear and makes sure everything is clean... My daughter makes sure everything is in the closet. But I believe I need to leave here.  Interviewer: Is your daughter well-informed... Do you tell your daughter what is still intended or do you notice that your daughter has already heard about it?  My daughter has already heard, she knows.  Interviewer: So, she can communicate well with the caregivers too?  Yes, my daughter knows. | Participant 14 |
|  |  | Well, the children are taking very good care of him, and my granddaughters, so he's being well looked after.  Interviewer: Yes. Is it nice for you to know that...  Yes, it's nice to know that, I hear, that he's being well looked after. They go to him every day, and I already had his food, so he has his warm meal. | Participant 21 |
|  |  | Interviewer: And were you able to ask your questions back then?  Yes, especially with my daughter. Especially with my daughter, I could talk to my doctor.  Interviewer: Yes.  Yes.  Interviewer: So, everything was clear in terms of information back then?  Yes, as a mother, not just as a mother, it's because it's how it is, I trust my daughter. If my daughter, I want my daughter to know everything. Because I know she will pass it on to my husband and my son. | Participant 19 |
|  |  | […] but other than that, I totally… I don't know. My daughter takes care of the laundry, and she makes sure things are clean […]. But I think I have to leave the hospital. -participant 14  “Your daughter seems to be well informed. Do you tell your daughter the ins- and outs? -interviewer  “No, she is contacted by the doctors. She already knows. I don’t need to tell her.” -participant 14  “Does the communication between her and the HPs go smoothy?” Interviewer  “Yes. My daughter knows | participant 14 |
|  | Subcode 6: **Professionals initiating decision-making with family instead of patient** | Yes... my daughter is involved in those matters... my daughter has agreed to the discharge...  Interviewer: So, all information is discussed with your daughter?  Yes, they should discuss it with my daughter as much as possible.  Interviewer: Do you like that it goes through your daughter and not through you?  Yes, because she also... she knows that I'm here and how everything is going with me, and she agrees with it. | Participant 7 |
|  |  | Interviewer: Explanation… So, were you told about the steps they were going to take?  Yes... [silence] to my niece...  Interviewer: To your niece...  Yes, he told everything to my niece... that it would be a long-standing issue. | Participant 2 |
|  |  | Interviewer: Do you tell yourself how it's going, or do you notice that the nurse or the doctor sometimes talks to your wife or children?  The time is much too short for that, we've only been here for a few days  Interviewer: yes  ..but the results were called by the surgeon to my wife, that's decent work, and I'm grateful to them for that. | Participant 18 |
|  |  | She is kept informed very nicely over the phone. About the surgery, about everything. Yes | Participant 15 |
|  |  | Interviewer: And are your children well-informed? Do they sometimes talk to the doctor?  Yes, my son said, I had him on the phone a little while ago, and I talked to that doctor for quite a while. He calls my son or my daughter. And he explains well, he says everything clearly.  Interviewer: Yes.  He even says things like, I didn't know that either... Then my son says that the doctor called him and talked with him for quite a while. Well, I think thats nice. | Participant 17 |
|  |  | Interviewer: So, she is very involved?  She is involved in everything, yes. I never knew, but they involve my wife in everything. | Participant 16 |
|  |  | Interviewer: Did the doctor speak directly to you? Was there family with you when you were admitted?  Yes.  Interviewer: Yes, and when the doctor spoke with you, was it with you or with your family?  The family was present, so yes. | Participant 13 |
|  | Subcode 7: R**especting patients** | And there I had, um... What I... I had a bit of the feeling that, um, as... Well, maybe I'm stating it too black and white... But, um, that you're a bit like, um, yeah, a bit... what's it called? Um... not seen as... fully qualified, let me put it that way... | Participant 5 |
|  |  | I mean, he's a doctor... I'm almost a concrete constructor... Should I put him in his place... that's not, um, not my job.... | Participant 7 |
|  |  | Interviewer: Yes... you believe decisions aren't discussed with you because the doctors think you're confused.  Yes... I think so, yes...  Interviewer: How did that affect you... your body language suggests you're a bit angry...  I was furious... I was in front of a man... well, what was he, actually? Well, he was a man, and he... an adult man, yes, you wouldn't think so, but he was an adult man... who said, "You need to leave now and hurry because you have to be here and there...Well, I decide that myself... he acted as if I were a 12-year-old boy, but it doesn't work like that... | Participant 7 |
|  |  | Interviewer: But you were washed yesterday, weren't you?  Yes, I did it myself... when the nurse comes in the morning... "Have you eaten yet?[in a nagging tone]... well, they damn well know that I don't eat in the morning because I have no appetite for food... and then it's, "When are you going to take a shower? I’ll decide that myself. | Participant 7 |
|  |  | Yes, I've been in the living room before... one says, "If you have to urinate a lot, you shouldn't drink so much...and then you get the other nurse... and she said I should drink more... imagine that... I think... I'll do what I want... | Participant 3 |
|  |  | Interviewer: And are your pain complaints taken seriously?  [silence] Pff, maybe one understands it, and the other doesn't... there's one of them, I do consider her a professional, I must say... "Sir, we'll both grab your legs and throw them overboard like this...I say, that won't work... yeah, you have to push through it for a moment because, um... that's not acceptable, you know... because I know best how far I can go myself. | Participant 8 |
|  |  | When it comes to pain, you had to indicate the level, from one to ten... what rate do you give for the pain, they would ask... I said eight and not lower... if they can do something with that, then it's fine... but when you're in pain, I can't feel it... you can tell me what you want, but if I don't feel it, I just have to listen to what I should do... handle it very gently and not too roughly... that way you have as little pain as possible...  Interviewer: Were they attentive to that?  Sometimes, yes, but you have to endure it for a moment, because otherwise, it becomes completely stiff... then I think, where do you get that wisdom? Every person feels their own body best... and how far they can go. | Participant 8 |
|  |  | Interviewer: I assume you have Oxynorm or Oxycodone?  Yes, something like that, that little thing.  Interviewer: A small blue pill?  Yes. That hasn't been discussed yet. In the beginning, I received it from time to time.  Interviewer: Was it on your own initiative that you asked for it afterward, or was it just brought to you?  No, it was given to me. I asked for it once at night... pain. "Do you have pain? No, not right now, but I was restless. Well, if you don't have pain, then you don't need to take those pills. Then they walked away. But those were a couple of nurses I had never seen before. They could be the night shift or something like that.  Interviewer: How did that make you feel when it was said to you like that?  Well, I actually felt a bit anxious, a bit like, why do you do that? So resolute, don't be... come on. | Participant 10 |
|  |  | Yes, they want attention. And then I think, don't treat me like an old person. I am old, I know that, of course. But I'm not whiny. I don't want to be, you know. | Participant 12 |
|  |  | Well, no, that's not how I experience it either. I mean, I'm old, sure. But they shouldn't treat me as if I'm a fool, so to speak. | Participant 12 |
|  |  | They all say "X [first name]and I really appreciate that, it's more personal | Participant 7 |
|  |  | Interviewer: Were your complaints taken seriously?  Yes, well... yes, absolutely... | Participant 6 |
|  |  | Um... the personal... they are aware that you're old... you explain what happened, and they also handle it very carefully... | Participant 8 |
|  |  | Then I came up here, and you have to get into the bed, and I have to say, 'guys, be careful, it hurts so much...' I'm just screaming because I can't hold it in... and I keep sighing deeply... but it's a connection between a patient and nurses."  *(also related to subcode 2.5)* | Participant 8 |
|  |  | Yes... in the end, you're an adult, not a little child, and when you indicate it, she should acknowledge it. | Participant 8 |
|  |  | That you're being listened to, that they take you seriously, and show respect | Participant 4 |
|  |  | No, he was just human. He was just as approachable as... Just like that young man who just left, I went for a walk with. Well, then I immediately feel at home.  Interviewer: So, it wasn't like the doctor is here, and you are here. You didn't feel that way.  No, no, I don't like that at all. I might be mean, but then I'd say, 'Oh, you have a cold potato in your mouth.' That's what we used to call it, that pretentiousness. No, I don't really like that.  *(also related to subcode 2.3)* | Participant 12 |
|  | Subcode 8: **Characteristics that enhance a professional patient-healthcare professional relationship** | And then I was in the, um, also in a room where he was. And he says, well, we're going to, um, do this and we're going to do that. In a friendly, quite friendly manner. And he did it, um, in a very pleasant way. And not in a, I'm the boss here, or I'm the doctor, and you, um... No, he explained it well. | Participant 5 |
|  |  | And he said, well, um... He told the doctor that I was doing well, and that, it's going in the right direction. And that gave me a good feeling again. | Participant 5 |
|  |  | Yes, also a male nurse said to me, ‘you can do it’ | Participant 10 |
|  |  | Yes, in the morning.. We were quite a bit tough at each other.  Interviewer: Did you like that?  Yes, I actually needed it too. Yes, a bit... come on, let's do it together. He was like, "Come on, let’s straight up for a moment." | Participant 10 |
|  |  | Yes, I still go to the toilet by myself, sometimes with assistance. But they do say, 'You're 91, and we don't see it that often,' so I'm a bit proud of that." | Participant 18 |
|  |  | She took her time for me, and no unpleasantness, nothing | Participant 7 |
|  |  | Yes, physiotherapy takes the time and provides good instructions. When I do the exercises, she says, 'Make sure to keep your toes and your leg straight and lift your toes up.' She's a tough lady... yes, she takes her time and gives good instructions. And she also puts you in your place, just like some of the nurses here. The physiotherapy has a strong voice and articulates clearly. | Participant 7 |
|  |  | People take their time for you... they prepare your plate, cut the food if you want... a dessert... no, I really have no complaints... otherwise, I would be lying." | Participant 3 |
|  |  | Um... the personal touch... they consider that you're old... you explain what happened, and they also handle it very carefully. | Participant 8 |
|  |  | That gentleman did it very well. He used all sorts of things... he used the railing, he used a little stand... so, eventually, I could stand... but he spent quite a bit of time, and what's the result: I took one step forward, nothing more. It just wasn't working | Participant 8 |
|  |  | It's a bit awkward to call a nurse for a glass of water at night, but they do it without grumbling... or they come to you and ask if they can do anything else for you. I liked it." | Participant 8 |
|  |  | Yes, certainly. It's not like they're constantly watching the clock, thinking, 'I need to go. | Participant 11 |
|  |  | Interviewer: What do you think of the living room?  Yes, I've been there, and I found it quite a pleasant change of environment... yes, and the lady who works there did a great job because she even painted and clipped my nails... yes, that was very nice... yes, they're not obligated to do it, but it is done... | Participant 4 |
|  |  | Yes, very good, and I can't say anything other than they're always ready for you. When I call, they just come. If I see one, I call out, and they come. | Participant 17 |
|  |  | For example, if I ask for a specific topping, it's never like, "Again?"  Interviewer: Yes.  It’s in order. Smooth. Well, I liked it  Interviewer: So, you can communicate well what you need, and they well responded?  Yes, really. When I need to use the restroom and press the button, the light is on in the nurses' station. Well, then they come and take you there. And when it's time, and I'm finished, I press the button in the restroom. Then they come again. And that's the whole process | Participant 16 |
|  |  | They have all the patience. It's not like she says, 'No, I can't really wait for you.' No, she goes through everything carefully | Participant 17 |
|  |  | No matter if someone is a doctor or a cleaner. I talk, and I don't make a distinction. You're all human, and I don't differentiate. | Participant 17 |
|  |  | Yes, yes. If they do something for me, I thank them. I say, you're very kind to me. And I appreciate it a lot. | Participant 14 |
|  |  | Oh, then we walk and chat for a while, checking how I'm doing and if there's anything that needs to be done. It's just [mentions first name]. And then I say, my name is [mentions name], but what's your name? | Participant 14 |
|  | Subcode 9**: The added value of humor and having a chat** | Interviewer: Were these kinds of things also discussed with you during your admission?  No | Participant 10 |
|  |  | They are all kind, but the care you really want for yourself.. you're not inclined to express that quickly.  Interviewer: Why not?  I think it only comes up when it weighs on your soul, when a conversation unfolds, when someone asks about you. Not like, good morning, is everything okay? Yes, yes. You see, then everything is fine. But if it becomes a conversation with a touch of intimacy, then I think you want to... I couldn't do anything about this either. | Participant 10 |
|  |  | I think that when I'm lying in bed, and they come in and start talking, it's more about being tough. You don't want to reveal everything right away, so when they come in all cheerful, you go along with it. | Participant 10 |
|  |  | No, no. I don't have any interest in that either. My husband and I were always together, so I have no desire for it at all. | Participant 11 |
|  |  | They are constantly busy, so I don't want to ask much. The real conversations I have with my family or, for example, my roommates.  Interviewer: Yes  I don't want to disturb them either. I already find it burdensome to ask for help putting on my socks.  *(also related to subcode 4 and 5)* | Participant 20 |
|  |  | Well, so, um, well, he [the physiotherapist] does it well. And now, um, even when I walk in the hallway...  Interviewer: Mmm.  As far as I can walk ... Um, always a chat or a quick, "How are you, Mr. X?" | Participant 5 |
|  |  | Interviewer: And the interaction... you say everyone is busy here... is there time for a chat?  No... hardly... that's not possible.  Interviewer: Would you have liked that?  Certainly...  Interviewer: And why would you have liked that?  Because then I can have a nice chat. | Participant 7 |
|  |  | Yes, yesterday, it was really nice. I spent some time talking with a lady. I think she's in charge there. Yeah, having a chat is nice. | Participant 7 |
|  |  | Well, okay... everyone greets each other, and maybe I don't know all the nurses yet... but they are all nice, and the one who was here just now... [the activities coordinator]... she specifically brought out a shuffleboard game... she does it very well, you know... | Participant 2 |
|  |  | Interviewer: What did the nurses do to make you feel at ease?  Just being there... I notice that [mumbles]... having a chat... just like that... they say hello for a moment... | Participant 6 |
|  |  | I enjoy people around me... and they don't even have to be directly related to me... at least, that's what I thought... but having some people around you... so you see something and can have a chat, that um... | Participant 1 |
|  |  | Humor is within you. Yes, it's a way to put things in perspective. And it's a way to connect with people... within an hour, I usually find a commonality with people. Then I know, oh, that person also has a dog... oh, that person has also been in a choir... you always look for points where you can talk together.  Interviewer: You specifically ask the nurses about that... you inquire about the person behind the white uniform.  For me, it's just a person... the white uniform means nothing to me... I am sometimes afraid... that there are nurses here for the uniform and not for the purpose they stand for.  *(also related to subcode 5)* | Participant 8 |
|  |  | Well, talk a bit with each other, you know.. It's, uh, and suddenly you notice that the person lives nearby and you've never seen them. Well, then you get into work history, that's usually what it's about. What you did in the past.  Interviewer: And with the nurses, for example, can you have a chat with them? Is there time and space for that?  Well, it's more like they usually have time, always friendly and helpful. But really having a chat with them, then you're keeping them from their work, that's not allowed at all. | Participant 18 |
|  |  | Interviewer: And speaking of talking, you need to do things for recovery, but in between, is there time for a chat and more?  Do you mean with the staff?  Yes. Certainly, yes. Not too long, but still. That you can chat a bit. | Participant 15 |
|  |  | No, it's going well, and they are really good. Very kind, all of them. I haven't experienced any unpleasant situations.  Interviewer: Yes.  And I like to have a little chat with them, but there isn't always time, of course. | Participant 17 |
|  |  | We make time, yes.  Interviewer: Yes, okay.  Yes, we do that every time. Yes, those people come by. Well, then we have a little chat,. | Participant 16 |
|  |  | Yes, it's different at the other ward.. It varies with each one. With some, you get the feeling that if they have helped you, it's good. And for others, you feel the need to have a chat.  *(also related to subcode 5)* | Participant 13 |
|  | Subcode 10: **Establishing trust in the professional patient-healthcare professional relationship** | Eh, they did ask, like, what kind of work did you do and, well, things like that. And then you get a completely different...  Interviewer: Yes, then you establish a different connection.  A completely different connection.  Interviewer: Yes.  And then you can also talk about it with each other in a good way... | Participant 5 |
|  |  | Nice. You get to know a bit more about me as a person like this... I think it’s beautiful. | Participant 1 |
|  |  | Interviewer: Is there attention given to the mental aspect? Feelings of sadness, anxiety?  No, there hasn't been any attention to something like that...  Interviewer: Would you have liked it if there was more attention for that?  Mental?  Interviewer: Yes...  Well, we are working on that now... having a good talk... yes, it's nice that they ask the patient... chit-chat... but I can never be serious... [silence] sometimes it needs to come out, you know... but I don't mind... | Participant 3 |
|  |  | You are actually the first one who... I happened to hear this week from one of the patients, also a lady who is here, she had lost her friend. That's a bit of a sad situation. We got into a conversation about change. Then, just like now, it came up... | Participant 10 |
|  |  | Oh, then we walk and talk for a while, checking how I'm doing and if there's anything that needs to be done. It's just [mentions first name]. And then I say, my name is [mentions name], but what's your name? | Participant 14 |
|  |  | Well, every time, for example, if I know.. I know.. the one who comes to get me out of bed is [name].  Interviewer: At nine o'clock. Yes?  And then at eleven o'clock, [name] or someone else from the team comes to help me back into bed...  Interviewer: They assist you back. Yes, and how do you find that assistance?  Well, good. For me, it's that contact. That's what I think. Contact, that's important, right? | Participant 19 |
| **Theme 3 – patients’ luggage** | | | |
| **1. Being a patient** | The notion ‘being a ‘good’ patient’, is about how patients think they should act while in hospital and the strategies they use to cope. Mainly, older patients rely on authority of healthcare professionals. This means that the loss of autonomy, control and independence is outweighed by the anticipated recovery. Patients may not like what is happening, but accept it if it means a quicker return to home. However, the strategy of acceptance can turn into one of submission/resignation if patients do not reassert some form of control (-moving to the passengerseat) over time. | |  |
|  |  | I don't know, because it's also very difficult these days, so you have little to say because of corona, I mean, it's really difficult. | Participant 11 |
|  |  | interviewer: yes... but if they say "we are going to wash you now and you actually don't want that... is there any discussion with you?  no... not really... but that's okay...  interviewer: no... why not?  why should it be?  interviewer: because your opinion is important! [laughs]  well, they also need to do their job so yes... [silence] | Participant 4 |
|  |  | You can't choose. No, but it is what it is. I have it.., it happened to me. | Participant 12 |
|  |  | I think I have an opinion, but it doesn't matter, does it? That's how it is. | Participant 15 |
|  |  | But still, it can't be ... If more people say all of this.., right? Who am I to say that I disagree? | Participant 15 |
|  |  | And then I think, can you do that? But it is what it is. | Participant 17 |
|  |  | You're here for a brief time. And about the surgery, you can't really talk about it; you have to undergo it. | Participant 20 |
|  |  | I think you just do what they ask of you. For example, when they said I would go to another department. Then I can't really say, no, I don't want that. | Participant 20 |
|  |  | No, you just go along with it. Just like with everything else. You just follow the treatment, and you also feel for yourself how it goes. Who am I to want to do it different? | Participant 20 |
|  |  | I don't express myself easily. | Participant 13 |
|  |  | Yes... because then they had to... they have the right people, of course.... | Participant 5 |
|  |  | They all did their very best.... That's for sure... | Participant 5 |
|  |  | And that um... I say [to his son], but you're not going to.., I warn you, ..don't complain [to the nurses]. Because I have nothing to complain about. | Participant 5 |
|  |  | Well, I know, it doesn't always get along between people. You have to take that into account too. | Participant 5 |
|  |  | But I must say, there were hectic moments.... | Participant 5 |
|  |  | No, because... she has to grab something for me while they're running around with bedpans and diapers... that girl doesn't know where to go first.... | Participant 7 |
|  |  | ... but we were received very well ... really... yes... | Participant 2 |
|  |  | ... and put in a cast... yes... we sat there for quite a while, but it was fine. The doctor still has to prepare everything, of course. The assistant also said it would take some time. | Participant 2 |
|  |  | Well, for example, um, yes... I have to think for a moment. a washcloth on my face... they really help me well. Only I have to wait sometimes... if someone is on the bedpan early in the morning. this morning too. yes, you have to be patient. | Participant 2 |
|  |  | Yes. I just think... yes, look... you have to listen... it's busy... you have to wait sometimes when you need to use the bathroom.... then you just have to be patient.... yes, that's what I think... it's not their fault.... but there's simply not enough staff, that's just how it is.... that's the case in all hospitals.. | Participant 2 |
|  |  | Yes, listen, in the morning too, I just wait until I'm helped. but listen, I can't expect to be helped right away... it's everywhere... not just with you... but everywhere they have a shortage of staff... | Participant 2 |
|  |  | A bit grumpy... but it doesn't matter... ` | Participant 3 |
|  |  | They're sometimes busy and then they say: we'll be with you shortly [silence] no... it's not that bad... but I say... | Participant 3 |
|  |  | It is what it is... I won't complain about every little thing, like that nurse is there for, I don't do that... really not... | Participant 8 |
|  |  | But I don't mind.... I like being among people.... | Participant 8 |
|  |  | That they listen to you.... that they take you seriously and show respect | Participant 4 |
|  |  | But well, you can't do anything about it | Participant 15 |
|  |  | I probably have an opinion but it doesn't matter, right? That's how it is. | Participant 15 |
|  |  | They're pretty nice people, they all do their best. Really. | Participant 15 |
|  |  | Well, pretty good, yes. But it's probably just me, you often have to wait a long time. If you've asked for something, like a bedpan or you have another question... But it's probably my own impatience. | Participant 13 |
|  | Subcode 2: **Using the call button** | Um, that I had to pee, and you didn't know where you, um... And that they also do it with a voice of, oh, he has to pee again, or something like that. | Participant 5 |
|  |  | And I also dare... Usually when I, um, have to pee and I can't find the stuff, or I don't know where it [the urinal] is, then I don't dare anymore, um... | Participant 5 |
|  |  | No, no... there are calls all the time... but when they call when something is really wrong, they don't come.... yeah, that's true | Participant 9 |
|  |  | But yes, then I say, yeah sorry, I didn't press the button, it was just... But they are never angry about that or anything, no. | Participant 11 |
|  |  | Yes, I had a bowel movement for the first time. Well, a bowel movement... It looks like it. At first, I would pull the string on the toilet so they would come help me. Later, they asked if I had pulled the emergency bell. Apparently, it’s an emergency bell.  Interviewer: Yes, indeed.  Since then, I don't do that anymore and go back to the room myself. | Participant 20 |
|  |  | I did have some trouble with that, but that was all in combination with, um, uncertainties... then, um, I come back to my peeing. You don't know, oh should I be there or can I call... and um... they don't make a fuss about it or, um, | Participant 5 |
|  |  | Yes, I did.. I was in the hospital once... then I had wet my pants.... I knew that if I called, a kind nurse would come... so I didn't call and when she later came into the room I said, go away... she asks what's wrong.... I say this and that.... and then she still helped me, but I felt so sorry... | Participant 7 |
|  |  | Yes, that... see, you know what it is... if I were to do it in my pants, then you would have more work, right?  Interviewer: yes, that's true  Then I think, yeah, I'll wait a bit. but yeah.... you might have shortages of staff.... that's everywhere, not just with you...  Interviewer: do you notice a lot of shortages?  No, not at all.... [silence] I just patiently wait until I get help... | Participant 2 |
|  |  | No. And then I also have to, a nurse has to go with me. So no, they doesn't have to be | Participant 21 |
|  |  | That's quite an undertaking... before you're in that wheelchair and then you have to go up and down again... that's a lot of work... | Participant 8 |
|  |  | But then I think why don't they do it themselves, that's what I mean... then I think yeah... they're already so busy, but yeah, then I have to bother them a bit, | Participant 8 |
|  |  | Then I felt that I had to go to the toilet. So, I, well lying down, you have to dangle your legs over the edge and that doesn't happen right away. And when I managed, I called, and they came. | Participant 18 |
|  |  | Yes, just barely, I think. It's really, in my opinion, there are really a shortage of staff. | Participant 15 |
|  | Subcode 3: **expressing illness or discomfort** | Yes, when I have pain, I get a painkiller. | Participant 2 |
|  |  | Interviewer: Yes... when you had pain.... did you then call the nurse for pain relief?  Yes... no... what can you do?  Interviewer: Um, extra pain medication... | Participant 6 |
|  |  | Interviewer: When you have pain, do you call the nurse?  Well, not always.. if I have a little pain, I don't call, but if it's severe, then yes... | Participant 9 |
|  |  | That's how it goes. I don't have much pain. | Participant 1 |
|  |  | Here it hurts. From above, everything is fine, but from below... the back... but yeah..., it's not that bad though... | Participant 3 |
|  |  | You should be happy that there is someone to help you. | Participant 8 |
|  |  | Okay, it hurts because, yeah... but otherwise... you are healthy, so yeah... | Participant 4 |
|  |  | Well, exercises... then you can sleep well afterward... | Participant 4 |
|  |  | No, why? I have it, don't I? She can't do anything about it. Yes, I could say.... They always ask you, do you have pain or is it okay? I say, well, it's okay. Do you want aspirin? I say, yes, I would like that. And then I get two aspirin. And I take them, of course. But then I think, hello. If you speak about my friend, and we're about the same age. Well, she takes eight aspirin a day. | Participant 12 |
|  |  | But I couldn't help people who complain. No.. you just have to be yourself. You shouldn't keep complaining and acting pitiful... And a lot of old people do that. | Participant 12 |
|  |  | And they've been through a lot, you can still feel it, with your thigh. That's why I have to keep my feet straight for that, I try to do that | Participant 18 |
|  |  | Yes, indeed, that's not bad. It's always like that at the beginning, it's still too stiff, and then a bit further, halfway, it's already better | Participant 18 |
|  |  | Pain, yes, God, which must be, yes. That woman [points to another patient in the room] had pain for six weeks. So, I'll just wait. But I'm doing my best. | Participant 15 |
|  |  | Yes, and then I did have pain, but yes. It's like that. | Participant 17 |
|  |  | Oh, that's okay, but walking hurts, of course. That's logic. | Participant 17 |
|  |  | In any case, I can stand again. Walk with a walker. But it does hurt. | Participant 16 |
|  |  | And when I start walking, it does hurt. But I really don't have to walk that fast. | Participant 16 |
|  |  | I'm not picky. | Participant 16 |
|  |  | No, I think it's my own fault. This helps me accept the situation. | Participant 20 |
|  |  | I just have to actually learn to walk again.  Interviewer: Yes because I also see a walker?  Yes, and they try to make me walk behind it. I do have to make sure it's unlocked. First, it has to stay closed.  Interviewer: Yes  And then the one who walks with me comes. And then we unlock it. And then um, this leg is good. And then I just try to walk.  Interviewer: And how is that? How do you find it yourself?  It's coming.. It's about to happen. | Participant 14 |
|  |  | Oh, you just have to endure it. Complaining doesn't help. Fortunately, I have still a good mental health. | Participant 14 |
|  |  | I wouldn't say, I think, I wouldn't say: tough luck!  Interviewer: Yes, that's how you see it? Yes.  Such as life.  Interviewer: Yes, well, we've been sitting for over an hour.  Yes, it was a nice talk. And my family will tell you: 'Be careful, she likes to talk!'  Interviewer: Well, that's a good trait, right? Better to ventilate than to bottle up.  Well, my trait has brought me very far. That's why I can give lectures at the university. I do a lot of games, the students often ask me: 'Miss [name], can you give us a game?'. Because I arrange games depending on what I think they need. And suppose, and I call that 'stretch', and a 'stretch', you know, to stretch. And a stretch is something, it should take you out of your comfort zone. And I'm here, I have a booklet and a pen. Just so, I see something and I think: 'Oh, that would be a nice stretch'. | Participant 19 |
|  | Subcode 3: **strategy of acceptance** | And those nurses last week... all they do is sigh. I tell them, I can't hold my water like a camel. (laughs) That nurse doesn't say anything back... no sense of humor. I say, they (patients) are all camels here... so I ask, don't you have a dessert? Those kinds of jokes... that's what I like. Just let it be. | Participant 3 |
|  |  | This early morning... then they had to take a lot of blood... you seem like a farmer, milking a cow... she says, "Well sir, it's a bit different. I always make jokes, you know... what's the point of being grumpy? Absolutely no point...  Interviewer: Is humor a way of dealing with things?  Humor is in you. Yes, it's a way to put things into perspective. And it's a way to connect with people. | Participant 8 |
|  |  | Interviewer: You like making jokes?  Yes, of course... But I tell you, it's not easy, especially because I can't walk. That's really tough. | Participant 11 |
|  |  | He said, "You have to go to the hospital. Then I said, "Can't you come up with something more cheerful?"  Interviewer: Exactly...  But yes, unfortunately, I was still taken to the hospital. | Participant 4 |
|  |  | So, I said, "Are you going to take me home?" | Participant 12 |
|  |  | Well, ... a little explanation beforehand would have been nice. Like, "Mr. X, we have a common room... they do this and that there... would you like to take a look? | Participant 5 |
|  |  | Sure, in this case... And well, actually, yesterday I felt bad about joining that common room activity. | Participant 5 |
|  |  | Well, I haven't seen them for two mornings now, so I just take the medication... | Participant 7 |
|  |  | Interviewer: Do you tell them then? ‘I want to do it myself?’  No... I shouldn't burden them too much... they have their routines. | Participant 7 |
|  |  | No, no... [silence] I just patiently wait until I get help... | Participant 2 |
|  |  | Yes, it can't be helped... they make it a bit cozy here in the living room... just sitting together... | Participant 2 |
|  |  | Yes, listen, in the morning, I just wait until I get help. But listen, I can't expect to be helped right away... it's everywhere... everywhere they're short on staff... | Participant 2 |
|  |  | No, because they pay attention here... you can sit at the table right away... | Participant 2 |
|  |  | Well, listen... I think it's good.... it must be good... | Participant 2 |
|  |  | Listen, I adapt... here at the table too... everyone is different... you shouldn't make it too difficult... | Participant 2 |
|  |  | Is there consultation with you, or do they only give information?  [silence] Well actually... no, I just went to the department, and then I’ll see... | Participant 6 |
|  |  | [silence] I really wouldn't know... but as I said, it just happens to you... and then you just go with it... I really wouldn't know... | Participant 6 |
|  |  | Interviewer: Yeah... we have quite a strict routine here... you wake up... wash, dress, breakfast, exercise with the physio... what do you think of that routine?  Well, that's convenient... they said the same thing here... there's physio only three times a week, and they found that a drawback... but can't you exercise on your own in between?  Interviewer: You can certainly exercise in between... do you see that as a personal responsibility to get better?  Yes, I do... you have to get better yourself, and you have to do it yourself. | Participant 6 |
|  |  | I think there are things that I can't easily do on my own, and if someone else takes over, I'm happy with that. | Participant 1 |
|  |  | Well, I wash myself completely on my own again... it wasn't allowed at first... | Participant 3 |
|  |  | Then I just surrender, and that doesn't bother me... [silence] | Participant 4 |
|  |  | Interviewer: Yes... but if they were to say "we're going to wash you now and you actually don't want that... is that in consultation with you?  No... not really... but it's not necessary...  Interviewer: No... why not?  Why yes?  Interviewer: Because your opinion is important! [laughs]  Well, they also have to do their job, so... [silence] | Participant 4 |
|  |  | Interviewer: And the routine of the department... we keep a tight rein here: you go to sleep, you're woken up in the morning, then you're washed and dressed, breakfast, and then exercise with the physio. What do you think of that routine?  I think I like that, yes. Can't point it out exactly, but I like it.  Interviewer: Why do you like it?  Maybe because it's a routine. | Participant 1 |
|  |  | Yes, good... [silence] I'm not difficult at all. | Participant 4 |
|  |  | But.. I'm very easygoing. I accept it. | Participant 12 |
|  |  | Actually, I'm not allowed to do anything without someone being present, you know, | Participant 18 |
|  |  | Certainly. Well, this morning she went into the bathroom with me. And she washes me, but I can wash my face myself. | Participant 17 |
|  |  | Yes, but I also want to do things myself, that's just how I am. So, I do some exercises. But I don't do much else yet, I don't dare. I'm always afraid that... If she says, do it, then I'll do it, but she always... if I walk she always stays with me. | Participant 17 |
|  |  | Interviewer: Yes because you're get help with washing yourself. How do you feel about that?  Oh, I think it's fine. No, that's good. And we do it together.  Interviewer: Together?  Yes, I wash the front of my body, she does the back. Everything is done within five minutes.  Interviewer: Is it being discussed with you, or is it being taken over continuously?  No, they don’t take over. | Participant 16 |
|  |  | No, no, no. And I don't mind that. And if I'm on the toilet and I'm done, and they let me wait for ten minutes... Well, whether I'm sitting here or there. It's the hospital. And those people (HPs) have to move on. | Participant 16 |
|  |  | Then I also got comments about that. But the older you get, the less you react to that. That's how it is.  Okay, so you accept everything quicker now?  Yes. | Participant 16 |
|  |  | I think you just do what's asked of you. For example, when they said I would go to another department. Then I can't say, no, I don't want that. | Participant 20 |
|  |  | No, you just go along with it. Like with everything else. You just follow the treatment and see for yourself how it goes. Who am I to want it differently? | Participant 20 |
|  |  | Yes, yes. If they do something for me, I thank them. I say, you are very kind to me. And I appreciate that very much. | Participant 14 |
|  |  | But I never complain. I never bother them with my concerns. | Participant 14 |
|  |  | I say, I can manage. I say, if you think something still needs to be washed, then... Or a male nurse does it.  Interviewer: Yes  I'm not shy about that. Those people have also learned to wash people. And they find it very normal. And I had to get used to it a bit, but I manage... If you have a doctor, it's also a man. | Participant 14 |
|  |  | I say God, well, what must be, must be. | Participant 14 |
|  |  | Oh, but I can get along well with anyone. I adapt very easily. | Participant 21 |
|  |  | If you have to wait for five minutes, it already seems like a lot, but when you see how busy they are...  Interviewer: Yes  Then you always think it's logical that they... that it takes a while. They are unable to come immediately or make their time available. | Participant 13 |
|  |  | Interviewer: So, if I understand you correctly, what you find most important is to know the names and times and such?  Yes.  Interviewer: How the daily schedule is… | Participant 19 |
|  |  | Interviewer: And the times are written down, from getting up, going to bed.  Yes.  Interviewer: Is that discussed with you, or is it written down for you? How does that work?  Written down, I ask for that. Yes.  Interviewer: And do they then coordinate with you: 'Shall we do it at such and such time?' or do you leave that to them?  Yes, yes, yes.  Interviewer: Yes? So, this is written down in consultation with you?  Yes, I like that. | Participant 19 |
|  |  | Yes. And when it was? Yesterday? And I said to them, 'I know you're busy, so I took one myself.' And tried to help a bit myself.  Interviewer: Yes. And what did they think of that?  They told me... And then I said, 'But my back still itches.' Then she said, 'Okay, let me have a look.' And she said, I said, 'You don't have to use your fingers.' And she said, she says: 'Use a wet glove.' And she said, 'Okay, come on. I'll help you. Come on!' And she did my side and my back.  Interviewer: Yes.  Everything is open for discussion. | Participant 19 |
|  | Subcode 4: **surrendering to the situation** | Yes, but, [silence] it’s not difficult for me... I just accept it... they do this, they do that, and I just accept it. | Participant 2 |
|  |  | Yes, I'll just wait and see... | Participant 6 |
|  |  | I just let it happen, I think... | Participant 6 |
|  |  | I can't exactly say. No…, I do know. The fact,.. that I am being cared for and that I am not alone... [silence] | Participant 1 |
|  |  | No, I don't know any of that, no... it’s unpleasant for me... because then I know nothing at all, and I just sit here and, well…, I'm fine with being helped and having to sit here... | Participant 1 |
|  |  | I think there are things that I can't easily do myself, and if someone else takes over, I'm happy with that. | Participant 1 |
|  |  | Well, I can't say that either. I don't do much myself anymore, so it doesn't happen anymore. Can I take a sip of water...? | Participant 1 |
|  |  | Interviewer: And does everything meet your expectations...  Yes... [silence] no... you just accept everything... | Participant 3 |
|  |  | Someone was supposed to come by, but I don't think he can do much. | Participant 11 |
|  |  | Yes, you had to walk, you know, over a little plank and this and that. But if you have no strength in your legs, well, then it stops. | Participant 11 |
|  |  | No, I think everything is going well. | Participant 11 |
|  |  | Everything is taken care of because I can’t do anything. | Participant 11 |
|  |  | Yes, I just let it all happen to me... | Participant 4 |
|  |  | It happened to me, that's very important. | Participant 18 |
|  |  | Um, I'll see how it goes. Yes  Interviewer: Yes, you'll see how it goes.  Yes, I don't have to work anymore so... | Participant 16 |
|  |  | Yes, but well, nobody can do anything about it. | Participant 21 |
|  |  | I simply give in | Participant 21 |
|  |  | No, I just let it all happen to me. | Participant 21 |
| **2. Healthcare professional identity** | Out of all healthcare professionals, nurses spent the most time with patients. Hence, nurses have great impact on how patients experience the received care. The interaction between patients and nurses is formed by perceptions patients and nurses have of one another. Patients described nurses as predominately young, sweet and female. Nurses were portrayed subservient to doctors and not as independent practitioners. Nurses took care of activities of daily living, questions about recovery and health or a chat should be saved for others. | | |
|  | Subcode 1: **Appearances of nurses** | They come quickly if I need help, but those girls (nurses) have to run like crazy, so I never push them... | Participant 7 |
|  |  | No, because... they have to grab things for me while running around with bedpans and diapers... that child (young nurse) doesn't know where to go first... | Participant 7 |
|  |  | I think it's for older people because they are naturally very tough. You don't just tell a young nurse... they are all young things, and they are all sweet. | Participant 10 |
|  | Subcode 2: **Focus on ADL** | But most of the social interactions I have is, of course, with those who bring the meals or those who washes me.. and checking the temperature, etc., and taking care of the medication. It's all taken care of, I understand. | Participant 18 |
|  |  | Interviewer: And the other way around, do they give you information about what you need to do?  Well, not much, of course. Because that's not really their task. They come to take care of you.  Interviewer: Yes  And there are indeed nurses who advise, 'You'd better do this or that when getting up,' while others simply assist without additional instructions.  *(also related to subcode 4)* | Participant 13 |
|  | Subcode 3: **Doctors handmaiden** | I said, ‘can you give me some advice?,’ and she said ‘yes’... I said, ‘you don't have to tell the doctor, but I can quickly deal with this.’ | Participant 7 |
|  |  | ..But they also see themselves mainly as a communication channel to the doctors… | Participant 20 |
|  |  | I know, yes... Doctor [name] told me: if I need something, I can always ask the nurse to call her (doctor).  *(also related to subcode 3.4.)* | Participant 19 |
|  |  | You're just a nurse too... (the patient said to the interviewer) | Participant 6 |
|  |  | ..that they immediately brought medicine when I asked for it... [laughs]  Interviewer: okay.... [laughs] --  But that's their profession, right? | Participant 6 |
|  |  | No, they stick a needle in my finger with all love and calmness and under a joke and such... The blood sugar was important for me. No, please, everything went... Everyone had their task.  Interviewer: It was very task-oriented?  Yes, of course. | Participant 10 |
|  |  | They are friendly; you can see that they know their profession. Then you quickly feel inclined to surrender because you can see whether it's a young nurse in training or a nurse who is certified and experienced.  Interviewer: How do you see?  You see it in the way they act.  Interviewer: Can you give an example?  The decisiveness. The feeling... Yes, the decisiveness and still friendly. | Participant 10 |
|  |  | That didn't work.  Interviewer: Have you been able to talk to them (nurses)?  Well, it's usually when they come for my blood tests. | Participant 21 |
|  | Subcode 4: **Conversing with patients is not part of nursing** | I thought, hey, I should ask the doctor why my medicine has changed... but I haven't seen the doctor at all. Well, I haven't seen him for two mornings now, so I just take my medicine...  Interviewer: And you haven't asked the nurse about your medicine?  No, I don't think her knowledge extends that far, I don't think so... | Participant 7 |
|  |  | They (HPs) are always friendly and helpful, but really having a chat with them.., then I keep them away from their work, and that's definitely not allowed. | Participant 18 |
|  |  | Interviewer: But if you have questions, then you would, if I understand correctly, ask the doctor rather than the nurse.  Yes, they (the doctors) are naturally more informed. | Participant 13 |
|  |  | Yes, that the difference.. Yes, that's also quite normal, of course.. That I talk easier to a doctor than to a nurse | Participant 13 |
| **Theme 4 – the Routeplanner** | | | |
|  | **1. The care pathway** | The care pathway describes how patients experienced the route travelled during their hospital admittance. | |
|  | Subcode 1: **Information and communication during hospital admittance** | Interviewer: And what kind of information did you receive in the emergency room?  Well.. for me as a patient, very little or nothing actually... because I was the victim, of course.  Interviewer: M hm.  And the communication was with the lady who was with me.  Interviewer: Okay, okay...  So, I had to communicate everything with her about my treatment. | Participant 5 |
|  |  | Interviewer: It wasn’t explained to you what they were going to do?  No, no, no... | Participant 5 |
|  |  | X... X... [patient's name] I kept hearing... X this, X that (outside the room of the patient). But it wasn't explained. ... But when the physiotherapist came, he explained it well...  *(also related to subcode 4.1)* | Participant 5 |
|  |  | Well, a bit of information beforehand would have been nice. Like... Mr. X, we have a common room... they do this and that there... would you like to take a look? | Participant 5 |
|  |  | Not in that sense, no. It’s not clear to me | Participant 5 |
|  |  | Interviewer: And here in the emergency room? Have you seen doctors who examined your hip?  No... well, a woman... I asked who she was, and she said the doctor, and then I thought, I'll just listen, but I don't trust it... | Participant 7 |
|  |  | No... it’s certainly not discussed with me. And why is that... I think they think.. [silence] he's crazy... (patient was in a delirium) we can't discuss anything with him... | Participant 7 |
|  |  | Interviewer: There hasn't been any information about your discharge?  Nothing, I know absolutely nothing. | Participant 7 |
|  |  | ..I think so, otherwise you won't get it... (nutrition drink)  Interviewer: No, have you ever asked why they give that to you?  Yes... they said everything you need in terms of food is in there...(nutrition drink) I don't know why, but you're also not allowed to drink the whole bottle at once, just small sips at a time...  Interviewer: Was that explained by the nurses? | Participant 7 |
|  |  | No... [silence] I don't know... it could be, but I'm not sure anymore... [silence] it might be... (about given information) | Participant 2 |
|  |  | Interviewer: I assume you spoke to the doctor’s downstairs (emergency room)... can you remember that?  No, I can't remember that... I think I went straight upstairs (the GTU)... I don't know...  Interviewer: Okay, so you don't remember exactly what was discussed... to get a surgery or not...  No, not at all... it just had to be... I had to go upstairs. | Participant 6 |
|  |  | Interviewer: Was it discussed with you beforehand that you would be taken out of bed immediately after the surgery and so on…?  No, actually not... I heard that from here [the roommates]... | Participant 6 |
|  |  | Interviewer: Was information discussed with you, or was only information given?  [silence] Well, actually... no, I just went to the ward, and then I'll see... | Participant 6 |
|  |  | Were you informed beforehand that you would have pain?  No... I didn't know... because the first night I thought.., oh, how intense, and later the doctor said ‘you'll have that pain for the first few days, but it will decrease... so I hoped for that... and now this morning it was a bit less... so I called again (at the nurse) and got another painkiller. | Participant 6 |
|  |  | Interviewer: And then you arrived in the emergency room by ambulance. What happened next?  I wouldn't know... | Participant 9 |
|  |  | Interviewer: A female doctor you said, and what did the female doctor say?  I wouldn't know... | Participant 9 |
|  |  | Interviewer: So, you are quite self-reliant... Can you give examples about the way you make it easier for yourself at the moment? Have you received information from healthcare professionals about that, or is it something you came up with yourself?  Well, I wouldn't know... | Participant 9 |
|  |  | Interviewer: And do you know anything about your discharge?  Discharge? No... I don't know anything.  Interviewer: Is it discussed with you, or is it discussed with your sister?  With my sister... she will know... I don't know... she will call because she decides for me what to do...  Interviewer: Do you find that reassuring, that everything goes by her?  If they tell me, I will forget it right away. | Participant 9 |
|  |  | Yes... I wonder about that too... I actually don't know... sometimes I'll ask the nurses something... I just don't know... that's the thing... yes. I don't know many things anymore... that I, no... I don't know... no... no... | Participant 1 |
|  |  | Interviewer: Did you get an explanation why you get that drink?  Yes... no... [silence] I don't like the drink.... | Participant 3 |
|  |  | I kept seeing people leaving the room, and I thought, why is that person staying away for so long... Well, then I got a wheelchair, and I thought I could ride a short distance at the ward, but I won't go through all the doors because maybe I'm not allowed to... And then I came here... [the living room] What a view,... Well, I had a really nice weekend. | Participant 8 |
|  |  | I don't know... because the doctor who wanders around, I never see him in the room talking to people... | Participant 8 |
|  |  | Interviewer: Did they tell what you could expect regarding pain?  No, I have no idea about my pain, and I'm sure.. I still take six paracetamols and I have some other ‘poisonous’ pill for which I have to take a drink. | Participant 10 |
|  |  | Interviewer: For example, issues like addiction and such.. Are these topics discussed with you?  No, no, that hasn't been discussed. | Participant 10 |
|  |  | Yeah, when I was downstairs (in the emergency room), you know... Everything went well. | Participant 10 |
|  |  | Yeah, I'm not... it's not in my nature to accept that they say... go to the living room… No.. | Participant 12 |
|  |  | The orthopedic surgeon conducted my operation, and the intention is that I will leave again. I am, of course, no longer interesting  Interviewer: ‘He has done his job’ (laughs)  Yes, yes, that's logical too. So, I'm afraid I'll go to [name rehabilitation] or I don't know where. | Participant 15 |
|  |  | Interviewer: Is there a central room where patients can come together?  Do you mean my wife and me?  Interviewer: No, here on the ward, is there a living room?  I'm not aware of that. It might be there, I think. | Participant 16 |
|  |  | No, I haven't seen people (HPs) from rehabilitation. But that will come.  Interviewer: Yes.  I'm alone and lonely. | Participant 14 |
|  |  | I feel very unfortunate because I had no idea that I had to go for rehabilitation. And then it turned out the facility is located upstairs (another department of the building). | Participant 14 |
|  |  | I thought rehabilitation center was far away from here (the GTU). I had no idea where it was. | Participant 14 |
|  |  | And then I was in the, um... also in a room where he (the doctor) was. And he said, well, we're going to, um... do this and do that, he talked to me in a pleasant and comfortable way. And he did, um, um... in a very pleasant manner. And not like, 'I'm the boss here or I'm, um, the doctor,' and you, um... No. He explained it well... | Participant 5 |
|  |  | Yes... I'll show the exercises I received [demonstrates]. And I received additional exercises from the very kind lady (physiotherapist)." | Participant 7 |
|  |  | Yes, it’s painful... especially that first night. Then, it gradually improved. I had cramps constantly, but last night it was better. I had cramps, but not as bad as before. And the doctor confirmed it, saying, 'That's true, you have painful cramps the first night, and then it gets better. | Participant 6 |
|  |  | Yes, they also said, um... 'the physiotherapist will come to you, and they...'. They only mentioned that nothing is broken, as shown in the x-rays, but something may be torn. However, the physio can help you. They will come tomorrow.' The physiotherapist was here and tried to walk with me. Well, I could take a step, with a lot of pain, and he (the physiotherapist) had to assess how it should proceed. He contacted the doctor again." | Participant 8 |
|  |  | Yes, because sitting is better for my hip.  Interviewer: So that was told to you?  Yes, they (HPs) told me that. | Participant 11 |
|  |  | No, no, because then I would have said 'can you do it in your own language then'. Because the doctor is also a human and Dutch, like me. No, I understood everything. | Participant 12 |
|  |  | Interviewer: How do you feel about the information that was given to you?  Well, a lot of the information was given in between the daily activities, you know…many things they still don't know, but in an hour they will know, about the control of heart rate, the blood pressure, of course. That is checked um all the time. Just before you came, they had just done that, temperature, and everything and anything.  Interviewer: They keep a close eye on you.  Yes, yes, yes. They know much more about me than I do.  Interviewer: Yes.  Oh, they (HPs) also ask a hundred times about my medication. Like, 'what are you taking?' Well, if you don't completely remember, it's like, it's a yellow pill or a brown pill. | Participant 18 |
|  |  | So, they (HPs) checked at various locations for rehabilitation. But yeah, they're all full. | Participant 15 |
|  |  | Look, there it is [points to an image of a hip with a surgical procedure]. Nicely broken, and then they put a pin through it.  Interviewer: That's a clear image. Was that clear to you back then, in terms of information? Was it explained to you clearly?  Yes, indeed. Especially with the help of the picture, which was incredibly helpful and clear. | Participant 16 |
|  | Subcode 2: **Waiting for the next step in the recovery process** | I found the wait for the surgery quite challenging, especially as I lay there waiting to be wheeled into the operating room. It took some time, and various thoughts crossed my mind. However, those feelings eventually subsided. You're aware of what's about to happen, so you can only hope that it happens a bit sooner | Participant 5 |
|  |  | Well, actually.... that it's a relatively short admission, I was actually surprised about that.... and where you go afterwards, well, you still don't know... and that's not nice.... the plan is that I can rehabilitate a quickly as possible so I can go home.... my husband is now in a care home because he can't be alone... | Participant 6 |
|  |  | I’m afraid for what will come next because I don’t know…. | Participant 1 |
|  |  | Whether I end up there [a rehab center] is still a question, as I could only if the hotel is full. So, I had to wait until Tuesday. I keep hearing Tuesday every time; I don't know anymore. That would only be known at 3 o'clock in the afternoon... | Participant 10 |
|  |  | I actually found it good [the admission]. When you're there [on the ward], you don't really notice it all. It just happens to you. Once I was allowed to sit in that wheelchair, get out, and walk, I thought, "I'll be discharged the day after tomorrow." ‬ | Participant 10 |
|  |  | Tomorrow afternoon, well, tomorrow morning at eleven, I will be transferred to [name rehabilitation center], a rehabilitation center, and I hope to be out of there within a few weeks. | Participant 18 |
|  |  | And I don't know at all how to proceed [after discharge], so I'll just wait and see. | Participant 18 |
|  |  | Called last night. Yeah, a very odd time, but it depends on the availability of spot in a home. So, they left me waiting. | Participant 18 |
|  |  | Yeah, and then in the hospital. Well, you just have to wait, right? That was on Friday, uh Saturday afternoon. What time was it? Yeah, around 1:30 or something. And, I had to fast, and then, I could have a photo taken. That's what they could operate on. | Participant 15 |
|  |  | There was quite a gap between the fall and the surgery, an entire day actually. And dealing with hunger during that time wasn't the most pleasant experience. | Participant 15 |
|  |  | So, I'm afraid I might have to go to [name of rehabilitation center] or who knows where. | Participant 15 |
|  |  | Waiting, waiting, waiting everywhere. It felt like it took forever. He [surgeon] said I would be operated on tonight but waiting for another 24 hours felt like a bit much. | Participant 15 |
|  |  | […] I have to go home because I'm done being examined, let it be. I don't need nursing care anymore. | Participant 17 |
|  |  | […] But it [discharge] will happen, of course, because, well, I'm only here for rehabilitation now.. | Participant 17 |
|  |  | I had surgery on Tuesday, and I've taken a short walk. But well, it's not much, actually. | Participant 17 |
|  |  | I stroll around a bit every now and then. But honestly, there's not much else to do here. Nothing at all, really. | Participant 16 |
|  |  | Nah, it's kind of a day-to-day thing. They could easily say, "No, it's not quite right; you need another day. Supposedly, I was meant to go home yesterday... not yesterday, but today. I was supposed to head home today. But then they said, "Hold on for another day." | Participant 16 |
|  |  | They could have easily sent me to a rehab center in [city name], but it's all booked up. Now, I have a physiotherapist coming to my room every day. Once I start the rehab, they'll spend about half an hour treating me each day, and then I'll be up and about again. | Participant 16 |
|  |  | Well, it won't be a walk in the park to move to another place. I don't think that's feasible. So, I'm hoping for a place with friendly folks and maybe some pleasant roommates.  Interviewer: That's indeed a waiting game.  Yeah, and you never know how much longer it will take. | Participant 20 |
|  |  | A hospital has fewer facilities for rehabilitation, but I understand that. A rehabilitation center is more specialized for that purpose and is therefore better equipped for it than a hospital. | Participant 20 |
|  |  | Yes, and that's why I'm waiting until I can be discharged. It can't be too quick because they also said if you leave earlier, it's not easy to come back if things aren't going well. | Participant 20 |
|  |  | It's all because of that darn hip. And then I have to rehabilitate at [mentions the name of the rehabilitation center], I had no idea what it was. | Participant 14 |
|  |  | Yes. Especially because I have to leave the hospital... [teary-eyed]  Interviewer: It affects you deeply, I see.  I have no idea what it [the rehabilitation center] looks like. And I also had no idea where it is. | Participant 14 |
|  |  | I thought the rehabilitation center was far away. I have no idea where it is. | Participant 14 |
|  |  | And this morning, I took a little stroll around here. yes, it went pretty well. So, I'm thinking of heading to a rehab center next week.  Interviewer: Alright, so the next move is rehabilitation?  Yeah, exactly. Because my 96-year-old husband can't really take care of me. He gets too emotional. So, no, that's not an option.  Interviewer: I see.  I wish it could have been different. | Participant 21 |
|  |  | That was so odd, then you don't get any help at all. They say, yes, for that, you need permission from the general practitioner first... | Participant 13 |
|  |  | [..] Actually, they can't do anything more for me here. The plan is actually that I, um... we had hoped to rehabilitate at [rehabilitation center]. I volunteer there. | Participant 13 |
|  |  | Well, I mean, this isn’t progressing either. I’m just sitting here. You go through the exercises, but you can feel for yourself that it isn’t going smoothly, of course. | Participant 13 |
|  | Subcode 3: **The efficiency of the care pathway** | Interviewer: Are people interested in getting to know you personally?  Not really, I don't think so. And to be honest, I don't find that very enjoyable. | Participant 1 |
|  |  | I have standard painkillers, and yesterday I called and told the nurse that I had such terrible pain... radiating down my leg... told them that i was in so much pain... and then she [the nurse] came back with a tablet... and I took it, and the pain was gone... it was really successful. | Participant 3 |
|  |  | They first wanted to know which medications I use and which doctor I have, and then followed a standard conversation. | Participant 10 |
|  |  | It went, whoosh. Throw a towel over it, quick and done. No fuss or... it was one patient after the other, as if there were four more in line…. It was like a bit of assembly line work. | Participant 10 |
|  |  | The operation went flawlessly. | Participant 10 |
|  |  | Yes, indeed. They all have respect for each other, and there is no loud talking or commotion. Everyone has their card; everyone has their patient file in their hand. It really runs like a well-oiled machine. | Participant 10 |
|  |  | I want them [HPs] to be engaged with me and my feelings. I want them to ask if I’m in pain en be interested in the answer… | Participant 4 |
|  |  | Interviewer: […] Have you had to fast for a long time, for example?  No, but that's not a problem for me. So, I don't really think about it, you know.  Interviewer: And are there things like complications and such? Have those kinds of things been discussed with you? Like, what to expect after the surgery?  I think everything has been discussed. | Participant 12 |
|  |  | […] No, why? I have it, don't I? She can't do anything about it. Yeah, see, I can say like, hey... They always ask you, do you have pain or is it okay? I say, well, it's okay. Do you want aspirin? I say, yes, I would like that. And then I get two aspirins. And I do take them, of course. | Participant 12 |
|  |  | That lady, she's not from the kitchen. Everything is picked up from the kitchen. I don't know if she's a trainee nurse, but definitely not from the kitchen.  Interviewer: Yes  She didn’t mention it [name and title] this morning. I find that unacceptable at any moment. Everything here needs to run smoothly 24 hours a day. So, that's my biggest annoyance.  Interviewer: Yes | Participant 15 |
|  |  | […] Yes, when they come to me, they do [know the reason for hospitalization]. They know what they're talking about, at least. It [the patient file] gets read and discussed. | Participant 15 |
|  |  | […] I said, oh no! But I received painkillers and such. Like last night, when you lie in bed for a long time, you get back pain, and then you get painkillers. | Participant 17 |
|  |  | No, generally, that's well known. Every day, a physiotherapist comes in. They take me for a walk.  Interviewer: Yes.  Every time they come, it's another step forward.  Interviewer: Yes, yes.  And he's a really nice guy. That makes a difference, you know.  Interviewer: And can he explain information well?  He can explain well, yes. But there's not much more to explain here. They've inserted a rod.  Interviewer: So, you don't really have many questions about it?  No, and there's not much they can tell about it. | Participant 16 |
|  |  | It seems like I have to express my concerns myself. I got the impression that the doctors were primarily focused on the recovery of the fracture and not so much on other aspects, like my lungs. I had to ask them myself to examine my lungs and check my hemoglobin levels. | Participant 20 |
|  |  | If I have questions, I just ask one of the nurses. But you know, everything seems to happen effortlessly. | Participant 21 |
|  |  | It all goes very smoothly, I find. During the day, you have the same nurses, and at night, there's a different night nurse. | Participant 21 |
| **2. The ward** | This code describes how patient experience the elements of care which make the co-managed care model unique | | |
|  | Subcode 1: **The livingroom** | I suggest, playing games or doing something together.... and now, with corona.... so, it's nice here.  Interviewer: Do you mean the living room?  Yeah.  Interviewer: You mentioned enjoying doing things on your own, but do the nurses also encourage you to do that?  Yeah... like I mentioned.... I can bring a nice cup of coffee to people in wheelchairs here.... I just put the coffee on the walker.... that way, you still have some interaction... | Participant 3 |
|  |  | Interviewer: You've been here for a few days now... what has really stuck with you... what should stay in your memory?  Stay in my memory? The living room... yesterday we were practicing... how to do it when it's Easter... you have to buy eggs, make two holes in them... and you have fried eggs, and you can paint them... and organize willow branches... where you can hang everything. They made this for spring, isn't it beautiful? I need to have people around me… | Participant 8 |
|  |  | Interviewer: What do you think of the living room?  Yeah, I've been there, and I found it to be a nice break... yeah, and that lady who works there did a wonderful job because she even painted and cut my nails... yeah, that was very nice... yeah, they're not obligated to do it, but they still do...  Interviewer: So, attention is paid to the patient...  Yes... | Participant 4 |
|  |  | Interviewer: What do you think of the living room?  Cozy. Yes, because when I was with that woman, she was there. I said, what a cozy space you have here, and the decorations along the edges are beautiful. Yes, she said, I'm really happy with it. Yes, we chatted about it. She asked, do you want some coffee? I asked, do I get that here too? Yeah, she said, of course! Well, we had coffee together. She said, will you come back tomorrow? I replied, sure, I'll come back tomorrow. | Participant 12 |
|  |  | […]No, that's something I can't handle on my own. But honestly, I'd prefer to move to a care facility now. You know, a place where I had a room, and a few communal areas. | Participant 12 |
|  |  | Yeah, you know, I was just thinking about it. Wondering if there's some sort of communal area or something. Nope, didn't have it in the previous place, and not here either.  Interviewer: Really? Were you aware that it exists?  Well, not really. Now that you mention it... Oh yeah, that's right. It wasn't there last time either, but hey, now that there's a TV, it's easy to get caught up watching it. | Participant 15 |
|  |  | Interviewer: Have you been out of the room lately?  Yes, yes. Well, not too far from the room. Just strolling around with the walker. Nothing more.  Interviewer: Yeah.  But I don't need to see much around here anymore. No. I'm fine as it is. | Participant 16 |
|  |  | Interviewer: Were you aware that there's a communal area?  Yeah, I know it's around, but I haven't checked it out.  Interviewer: No? Can I ask why?  Not really any particular reason. Just hasn't crossed my mind.  Interviewer: Got it. Would you be interested in going?  Not really. It's not like I'm avoiding people or anything.  Interviewer: Fair enough.  I'm just content staying here, you know? | Participant 13 |
|  | Subcode 2: **The patient rooms** | Interviewer: How do you like being in a room with four people?  Oh, it's kind of nice, you know.  Interviewer: Yeah, wouldn't you rather have a room all to yourself?  Nah, not really. | Participant 6 |
|  |  | Interviewer: So, you'd rather have some company in the room than be on your own?  Yeah, I guess... [pause] yeah, I think so.  Interviewer: Because of the social aspect?  Exactly, just for the company, you know. Of course, it depends on the people [laughs]. | Participant 1 |
|  |  | Interviewer: you share the room with another person. Would you have preferred a single room?  No… I don’t mind at all because I now know that he used to be…  Interviewer: your find the interaction enjoyable?  It’s just the space… the space is too small for two beds…. You have to squeeze through, and you can’t even fit a chair in between…. But I don’t mind. I like being around people. | Participant 8 |
|  |  | Well, I'm not too bothered, really. Sleeping alone wouldn't be my preference either. But if I'm sharing a bed with someone, I'm okay with it—it just depends on the company. I wouldn't want some old nagger beside me. But as my kids say, "No way, mom, you're still young yourself….." | Participant 12 |
|  |  | I think it’s going quite well; you know. Having a single room…. On the one hand, you have more privacy, but on the other hand, you're alone. I asked the staff if the door could be left open day and night. Then I can witness the activity in the hallway…. I think I would have preferred a roommate. | Participant 15 |
|  |  | I didn't like lying here alone at first, but now, well... Now, if the door is closed, I might ask to have it open. The nurse might walk by or she might come in for a bit. I would like that. | Participant 16 |
|  |  | I tend to do more when I connect with roommates.  Interviewer: So, if I understand correctly, it depends on who your roommates are for you to pass the time. If there are patients you get along with, you're more inclined to do more?  Yeah, that's right. I'm more likely to sit at the table and talk. There were also two American roommates who were discharged yesterday. One had a broken leg but was also paralyzed in one arm. | Participant 20 |
|  |  | I was truly amazed watching him handle things by himself; it was quite impressive. For example, he put on a sweater by holding the neckline between his teeth, looping the sleeve around with his other arm, and smoothly pulling the sweater over his head. He did it with great skill, and I found it really impressive.  Interviewer: That does sound impressive. So, you observed closely to pick up some tricks?  Yes, then you realize what you're capable of. At first, I couldn't put on my pants by myself. Bending to pull them up was impossible due to the pain, especially in my ribs."  Interviewer: yes.  but I'm the type who still wants to do it myself, so I'll try different things until it works. Today, I managed to get my pants over my ankle by using my other foot, specifically my big toe. That worked. | Participant 20 |
|  |  | [..] No. And why? Because I know, I can always get help, not just help, but talk to someone I know. If that weren’t the case, it would be a problem.  Interviewer: Yeah, and do you mean from your own family and friends or from...  From here.  Interviewer: ...from here.  I know the people [the nurses]. And they know me too. | Participant 19 |
|  | Subcode 3: **ADL support** | Interviewer: Alright. Were they interested in what you wanted? I mean, there are different ways to handle things. Did you get a say in it?  No. | Participant 5 |
|  |  | The nurses take good care of me. | Participant 7 |
|  |  | Interviewer: Are they inquiring about what you can or cannot do here, or do they just take over the care?  Everything gets taken over.  Interviewer: Do you like that?  No, because then I miss having that sense of independence... | Participant 7 |
|  |  | Interviewer: How much do you handle yourself when it comes to washing and getting dressed, or do the nurses handle everything?  Well, I prefer it when they let me do some things on my own... | Participant 2 |
|  |  | No, it's [ADL activities] taken care of... well... I get a washcloth to wash my face... [laughs]  Interviewer: Are preferences discussed? Can you, for example, take a shower? Is that an option?  Well... I don't feel the need for that yet. | Participant 6 |
|  |  | interviewer: You say you're not really involved in the care... you're not seen as a partner in care... [patient interrupts]  No... not as a partner... really [silence] it's done... and I think... listen... maybe it's because I have a bit of an understanding of how it is... they think, well, if she thinks or says this or that, it must be like this or that... that makes a difference... they said earlier... ah, you should have actually been in hospital... | Participant 6 |
|  |  | […] at home, I have a shower chair to sit on... no, I won't say, "This is how I want it, and this is how it should be done. The nurses know best. | Participant 9 |
|  |  | Interviewer: […] Yes, exactly... and by the nurses... are you encouraged to do things yourself?  No! | Participant 3 |
|  |  | [..] No, I had no say in the matter. They [the HPs] arranged something for me after discharge and the told me that will be going to that place. | Participant 8 |
|  |  | No, they [ the nurses] were really considerate... they know the situation, and there was this nurse who quickly brought the wipes [wash cloths]... "Is this okay for you, sir? And I told her, "You're doing a great job."  Interviewer: Were you ever asked about what you could manage on your own... did they motivate self-care?  No, but I told them, "Hey, I got this covered” | Participant 8 |
|  |  | Yeah, they didn't throw me into the deep end, you know. They showed me exactly how to do it. The first time, I was like, "Hey...but then I just did it too gently. It should be like, plop, in it goes. | Participant 10 |
|  |  | Self-reliance was not emphasized in hospital. The nurses decided what, where and how things were done. | Participant 10 |
|  |  | The nurses took it upon themselves to decide how I was going to be washed... | Participant 4 |
|  |  | [..] Well, I reckon they did it [ADL activities] for me because of the pain, you know. That's the thing.  Interviewer: Yes, but they didn't ask you what you'd prefer?  Nah, they didn't ask. But I would've said no if they wanted me to walk to the shower, for instance. | Participant 12 |
|  |  | They want you to do it [ADL activities] by yourself as quickly as possible. | Participant 15 |
|  |  | This morning the nurse assisted me in the shower. She washed my body, but I was allowed to wash my own face. | Participant 17 |
|  |  | Well, they [the nurses’ attempt to take charge. I'm like, just let me handle it... We've got a shower and a toilet for us patients here. | Participant 14 |
|  |  | So, that's it, right? Yeah, I’ll try to keep my spirits up, because I can complain oh, it's difficult and all. But that doesn't help either. Besides, they take good care of you. So, you know. | Participant 21 |
|  |  | Well, sometimes, before you even know it, they've [the nurses] already taken care of it [ADL activities].  Interviewer: [repeats the participant] They've done it again, yeah. And how do you feel about that?  Oh, I'd rather do it myself. | Participant 13 |
|  |  | […] And there are definitely some [nurses] who say, when you get up, you'd better do it this way or that [supply information]. And others help you a bit and that's it. | Participant 13 |
|  |  | Yes, they [the nurses] basically tell you what you need to do [during ADL] | Participant 13 |
|  |  | […] And freshening up? Do you do it yourself or do you get a hand?  I get some help with that. They even have these warm washcloths heated up in the microwave. | Participant 19 |
|  |  | Occupational therapy, yeah. Never gave it a shot before. It was just a thought in my mind, and then I thought, well, maybe instead of the walker, he could try a wheelchair. I used to be in a wheelchair years ago. So, I could, you know, move around on my own.  Interviewer: So, they're working with your ideas, from what I gather.  Oh, absolutely! | Participant 19 |
|  | Subcode 4: **Having to wait** | It turned out, when I came in here... it was very quiet. It was so quiet on that Sunday at the emergency room....  Interviewer: Were you helped right away?  Yes. Well, that did take a little bit, um...  Interviewer: It took a while?  Yeah... because they had to, um... they had to get the right people in, of course. | Participant 5 |
|  |  | Well, for example, um, yeah... I need a moment to think. A washcloth on my face... nothing too demanding, you know... no, they do help me quite well. It's just that sometimes I have to wait... like when someone is using the bathroom early in the morning. Happened this morning too. Yes, you need to have patience for that. | Participant 2 |
|  |  | Well, you know, "long is a relative term, and I've got plenty of patience. Can't really say.  Interviewer: Did it feel like a long wait to you? Did you feel like you were waiting for a while?  Nah, not really. I figure those folks have their own schedule. Yeah, what kind of schedule do they even have, you know? So, I'll just go with the flow and see where things go. | Participant 12 |
|  |  | That guy has to handle everything by himself all day, and it's just not manageable. That's the reality of it. And, well, what can we do about it? It's not our concern. Yeah, so...  Interviewer: How do you notice that there's a shortage of staff?  Well, you know, it's Friday. There's only one nurse around. That's just not right. And it was the same story at the beginning of the week. | Participant 15 |
|  |  | […] they didn't really know what to do with me. So, they just put me in a single room with the bed against the wall, so I couldn't get out. | Participant 15 |
|  |  | […] Well, it's alright, you know. However, I tend to wait a while, especially if I've asked for something like a bedpan or have a different question. But I guess it's just me being a bit impatient. | Participant 13 |
|  |  | They try to come quickly, but those girls [the nurses] have to run like crazy, that's why I never press the bell. | Participant 7 |
|  |  | […] Yes, they are busy. And then I think, let me spare that girl... I'd rather they take a coffee break than relieve me from a dirty diaper... because that's also dirty. | Participant 7 |
|  |  | No, because... she [the nurse] has to fetch things for me while she’s running around with bedpans and diapers front, left en center... she doesn't know where to go first. | Participant 7 |
|  |  | […] It has been put in plaster... yes... we've been here for quite a while, but it was fine. The doctor still needs to get everything ready, of course. The assistant also said that it would take a while. | Participant 2 |
|  |  | Yeah, listen, in the mornings too, I just wait until I get help. But you know, I can't expect to be helped right away. It's everywhere, not just here, but everywhere they're short on staff... | Participant 2 |
|  |  | […] Certainly... [pause] yes, absolutely. That's how I feel. They're all putting in their best efforts, always on the go, especially in the mornings... | Participant 2 |
|  |  | They are busy sometimes, and then they say, 'We'll be with you shortly' [silence] No... it's not that bad... but I say... | Participant 3 |
|  |  | I often find myself wondering why they [the nurses] don't take the initiative by asking if we need something else before leaving, you know? I mean, I get that they're really busy, but sometimes I feel like I have to reach out and bother them anyway. | Participant 8 |
|  |  | […] maybe there just not interested [in the feelings of patients]; they [HPs] already have to listen to so much, and they’re busy. I always assume that they are very busy. | Participant 8 |
|  |  | No, there is no time to attend to feelings of patients, for they [HPs] a really very busy. | Participant 10 |
|  |  | No, because they do make time for me. I can't really say they're too busy, as they genuinely take the time. | Participant 11 |
|  |  | Yeah, but I think that's reasonable. When I make a call, it's usually for important things, and they do come. I say, "Hello, and they mention, "Yes, you had to wait a bit. I respond, "Wait, come on. You all are too busy. They’re really busy, you know. | Participant 12 |
|  |  | There are too few staffed | Participant 18 |
|  |  | Then, I did feel that I needed to use the toilet (Interviewer: yes), so, well, from a lying position, you have to swing your legs over the edge (Interviewer: mm), and that doesn't happen right away. And when I managed to do it, I called, and they came right away. | Participant 18 |
|  |  | And those [nurses], they are so busy... the physiotherapy, they require ten times more time per patient. I get the impression that more attention is given to other patients. With me, they briefly go to the toilet, then just point out that I'm walking incorrectly, and that's the extent of it. | Participant 18 |
|  |  | Well, everyone is making an effort. It's just that there's a shortage of staff. | Participant 15 |
|  |  | Well, everyone is doing their best. It's just that there's not enough staff.  Interviewer: Oh, really?  That gentleman has to handle everything by himself all day long. And it's not feasible. That's just the way it is. | Participant 15 |
|  |  | Well, you know... When you call, the wait is quite long. Even if you call in the middle of the night when there's absolutely nothing happening, at least from what I can observe here.  Interviewer: Yes.  I can't see other wards, but I do think they could improve on that.  Interviewer: Yes. And you're referring to the long waiting time?  Yes, indeed. I called earlier this week, and it took at least twenty minutes for someone to come, even in the middle of the night. I can't imagine they're that busy. | Participant 15 |
|  |  | […] Yes, and then in the hospital. Well, you just have to wait, right? It was on Friday, um, Saturday afternoon. What time was it? Yeah, around 1:30 or so. I had to fast, and then I could have a photo taken. That's how they could determine whether I needed surgery. | Participant 15 |
|  |  | [..] well, just barely in my opinion. I truly believe there a few too few people [HPs] on the ward. | Participant 15 |
|  |  | honestly, I would really like to stay for an extra week [in hospital] for rehabilitation purposes, but I don't think that's possible, right? The rooms and everything are already occupied. | Participant 17 |
|  |  | No, I'm satisfied, and they [HPs] are really good. Very kind, all of them. I haven't heard a wrong word yet.  Interviewer: Yes.  And I do like to chat with them [HPs], but there isn't always time, of course. | Participant 17 |
|  |  | It used to be two of us, and now there are four [patients]. Sometimes, it does feel a bit hectic. It goes on like this the whole day. | Participant 16 |
|  |  | No, I don't mind at all. Even when I'm in the restroom and finished, if they make me wait for about 10 minutes, it's okay. Whether I'm waiting here or there, it's a hospital, and the staff have their duties to attend to. | Participant 16 |
|  |  | Well, they’re [the nurses] putting in effort to help me as quickly as possible, that’s true. But they only have two hands. | Participant 14 |
|  |  | We [the nurses and I] communicate well with each other and all that | Participant 21 |
|  |  | Have you seen the hustle and bustle here [on the ward’]? | Participant 13 |
|  |  | What matters most is that I can ring the bell. It's not guaranteed that they will always come, but I understand if they're occupied. | Participant 19 |
| **3. The Team** | This code describes how the healthcare professionals working in the co-managed care model/GTU ward function as a whole and how they are perceived by patients. Despite the differences in uniform, patients cannot distinguish between different professionals. | | |
|  | Subcode 1: **Distinguishing features of healthcare professionals** | I was furious... I was in front of a man... yes, what was he actually? Well, he was a man, and um... an adult man, yes, you wouldn't think so, but he was an adult man. | Participant 7 |
|  |  | The doctors, what did I want to ask them again... Yesterday I said, ‘is there a doctor here?’.... yes, the doctor comes once a day in the morning, well, I still haven't seen a doctor.... well, I'll save the questions for later. | Participant 7 |
|  |  | But my niece was with me, and um, we went straight to a doctor... who it was... I don't know... but we went straight... but, that doctor said: ‘she'll have to stay in the hospital because it's a serious fracture...’ it was completely broken here [points to arm] and um, which was broken. | Participant 2 |
|  |  | Yes, I think so, but I don't know them all... | Participant 2 |
|  |  | Yes... for example, the young man, who was there this morning, is he a doctor or is he serving food?  Interviewer: We have a young doctor around...  Oh, I only saw a doctor very briefly... I believe on the first day or so... but very in the distance... so | Participant 2 |
|  |  | I wouldn't know... they all look the same... and I can't remember so well anymore... I've already said.... they should write it down.... | Participant 9 |
|  |  | I actually don't know who a doctor was and who wasn't... | Participant 3 |
|  |  | Uh, those guys who... who conducted the X-ray and left... yes, we're going to discuss with the doctor what we're going to do...  Interviewer: but the doctor himself.. you didn’t see him at your bedside?  Uh, no... | Participant 8 |
|  |  | Interviewer: Did you see him often? Did you see him daily?  I saw him twice. | Participant 10 |
|  |  | Interviewer: But can you tell the difference, who is who?  No, not really, and I don't think it’s necessary | Participant 11 |
|  |  | I haven't seen them at all.  Interviewer: you haven't seen them at all?  Because he came in. I said, oh, are you the doctor? He said, no, I'm not a doctor, I do not have such a high status. I said, then I don't have to address you formally. No, just kidding. No, I haven't seen him yet. | Participant 12 |
|  |  | Well, I haven't spoken to the same doctor yet. | Participant 18 |
|  |  | I have seen a doctor, yes. But I haven’t seen my surgeon here. | Participant 15 |
|  |  | That lady, she's not the nutritional assistant. She brings the food and then collects the plates afterward. I don’t know if she is a student nurse but she is definitely not a nutritional assistant. | Participant 15 |
|  |  | Yes. But I can't see the difference between a nurse and... this morning she was here, the doctor... In the past, you had the doctor, and he was a dignified man. But now, all girls can become doctors. Yes, or girls, or women. Yes, very good, I don't say anything about it. But I can't tell if you're a doctor or a nurse. Yes, you (the interviewer) don’t even wear white clothes yet. | Participant 17 |
|  |  | It's just that you have to get used to it. Then she walks in the room and then another walks in the room. | Participant 16 |
|  |  | I don't know if he's a doctor or a nurse. They all walk around with a face mask. And that makes it difficult. | Participant 16 |
|  |  | For older people, I believe, as you're evidently quite resilient. You wouldn't just casually share your concerns with such a young nurse... they're all young individuals, and they're all pleasant, but you might not readily express the level of care you have for yourself. | Participant 10 |
|  |  | I said, at that age, I don't know your age, but I can guess. Are you already a doctor? He says, yes, I say, well done. Yes, very nice. | Participant 12 |
|  |  | Then two people came, and they were talking like that, but I didn't realize they were doctors. Then he said, yes, and then we're going to do this... So, I look at him. I say, you're not going to tell me you're a doctor. He says, yes. I say, how did you do that? Because you're so young? I say, well, then you're really, huh, a rarity. He says, no, there are more. Well, I was surprised that they become doctors at such a young age. Well, then I take my hat off for you. | Participant 12 |
|  |  | Now I have a young doctor. I really have to get used to it. | Participant 21 |
|  |  | Yes, that's a difference. Yes, that's also quite normal, of course. That you talk more easily with a doctor than with a nurse. | Participant 13 |
|  | Subcode 2: **Interdisciplinary communication** | They know what I have and.. | Participant 2 |
|  |  | No... because, for example... I'm leaving today and they all know that I'm leaving, where I'm going... | Participant 8 |
|  |  | Yes, indeed. They all have respect for each other, and there's no loud talking or acting. Everyone has their staff card, everyone has their patient file in their hand. It really runs like a machine. | Participant 10 |
|  |  | Interviewer: Do the nurses know what happened, or do you have to tell your story again every day?  No... [silence] they, uh, remember it pretty well... [laughs] [silence] | Participant 4 |
|  |  | Interviewer: Several times a day, different nurses come by. Do they communicate well with each other? Do you get the impression that they do?  Yes, I think so because they know what I have.  Interviewer: Do they know what you have, and you don't think like, Nurse A says this, and Nurse B says that...?  No, no. I think that they... Because I said it the other week, I said, you are very well informed. | Participant 12 |
|  |  | Oh, you have to check your medication chart a hundred times. | Participant 18 |
|  |  | Yes, when they come to me, they do. They know what they're talking about, at least. It's read and discussed with each other. | Participant 15 |
|  |  | Well, they are aware, but in the first days, I still had to tell how I got it. They naturally don't know. Yes, if I had just gone for a walk and then tripped and fell, well. But yes, this is a special case [laughs]. | Participant 17 |
|  |  | No, generally, that's known. Every day, a physiotherapist comes. Because he takes me for a walk.  Interviewer: Yes  .  Every time he comes, it's a step further.  Interviewer: Yes, yes.  And he's a really nice guy. And that makes a difference.  Interviewer: And can he explain things well in terms of information?  He can explain it well, yes. But there's not much more to explain here. A pen is inserted.  Interviewer: So, you don't have many questions about it?  No, and they can't tell much about it.  *(also related to subcode 2, theme 4)* | Participant 16 |
|  |  | Yes, it all goes automatically, I think. You have the same nurses during the day, and then you have a different nurse at night... But she came right away... I had a catheter, so I had to.. | Participant 21 |
|  |  | No, they know exactly.., even the next one, I think: oh, how do they know that? | Participant 21 |
|  | Subcode 3: **Team Spirit in the GTU** | You all know each other, and that's nice... they know what I have and .. | Participant 2 |
|  |  | Well, good... everyone greets each other, and I might not know all the nurses yet... but they're nice, and the one who was here just now... [the activities coordinator]... she specially brought out the shuffleboard... she does it very good... | Participant 2 |
|  |  | I've chatted a lot... this afternoon someone is retiring here.... then there's a party and water will be thrown... that happened yesterday too. | Participant 3 |
|  |  | Absolutely.... that makes it nice to work on the ward. | Participant 3 |
|  |  | You wake up, and then two nurses come in and the night shift, for example... they just treat each other normally... | Participant 8 |
|  |  | I have talked and had fun with the nurses. | Participant 10 |
|  |  | Interviewer: How do you notice that it's pleasant?  Just, they are nice on their own too. You know, not so, not too serious or anything. There's a bit of laughter. | Participant 11 |
|  | Subcode 4: **Difference in attitude and communication style between healthcare professionals** | And, when I had to pee, and you didn't know where you can find the bathroom... And then they do it with a tone of voice like, oh, he has to pee again, or something. | Participant 5 |
|  |  | It depends on who you get. | Participant 5 |
|  |  | Yes, the physio takes the time... and gives clear instructions... when I do the exercises, she says yes, and lift your toes and keep your leg straight, and toes up... she's a tough woman... yes, she takes the time and gives good instructions... and she puts you in your place, just like some nurses here. The physio has a loud voice and articulates clearly. | Participant 7 |
|  |  | Well, that you take good care of your patients and make it enjoyable...  Interviewer: Yeah... and has that expectation been met?  Well, some are more enjoyable than others... but that's everywhere. | Participant 6 |
|  |  | Yes... I've been there [living room]... one says... if you have to pee a lot, then you shouldn't drink so much.... and then you get another nurse... and she said that I should drink more... imagine that. | Participant 3 |
|  |  | ... and just sighing... I say I can't put a cork on it either... that nurse doesn't say anything back... no humor.  (a situation when the patient had to go to the toilet) | Participant 3 |
|  |  | Yes, yes.... yes... one is more willing to help than the other... | Participant 3 |
|  |  | I have a different nurse every day, and one is nicer than the other... but let it go and move on... they all do their best... it's the characters of people... some click with you, and others don't... | Participant |
|  |  | I came up here, and then you have to get on the bed, and then I have to indicate guys be careful, it hurts so much... I just scream because I can't hold it... and sigh deeply and just sigh... but that's a feeling between a patient and a nurse... | Participant 8 |
|  |  | she was a little bit upset | Participant 8 |
|  |  | [silence] pff, maybe one understands it, and the other doesn't... | Participant 8 |
|  |  | But that I messed it up... | Participant 10 |
|  |  | Interviewer: I assume you have had Oxynorm or Oxycodone?  Yes, something like that, that little thing.  Interviewer: A small blue pill.  Yes. It hasn't been discussed yet. In the beginning, I got one every time.  Interviewer: Did you ask for it, or was it just given to you?  No, I got it. I asked for it once at night. ...? ..pain. 'Do you have pain?' 'No, not right now, but I was restless.' 'Well, if you don't have pain, then you don't need to take those pills.' Then they walked away. But that was a pair of nurses I had never seen before. That could be the night shift or something like that.  Interviewer: What did that do to you when it was said to you like that?  Well, I actually felt a bit anxious, a bit like, why do you do that? So resolute, don't be ... come on. | Participant 10 |
|  |  | [silence] there's a lot of difference between nurses... | Participant 4 |
|  |  | Interviewer: But do you notice a difference...?  Yes, oh, it happened once, it's nothing.  Interviewer: Can you see the difference between nurses, physio, doctors? Because everyone walks in a white suit with a face mask on? | Participant 18 |
|  |  | And there are really some who say, 'with getting up, you better do this or that.' And others who help you and that's it. | Participant 13 |
