# Supplementary material for: Exploring geriatric trauma unit experiences through patients’ eyes: a qualitative study
Source: BMC Geriatr. 2024 May 30;24:476. doi: 10.1186/s12877-024-05023-z (PMC11140891; doi:10.1186/s12877-024-05023-z)
Supplement: Supplementary file 2 — Supplementary Material 2 [file 12877_2024_5023_MOESM2_ESM.docx]

| Table 1. Terms under which the co-managed care is known | | |
| --- | --- | --- |
| **Name** | **Author** | **Country** |
| Geriatric trauma institute | DeLa’O et al., 2014 (1) | USA |
| Rochester Model | Kates et al., 2010 (2) | USA |
| Geriatric trauma Services | Lorenzo et al., 2005 (3), 2020 (4)  Leede et al., 2020 (5) | USA |
| Geriatric Trauma Unit “G-60” | Mangram et al., 2011 (6), 2012 (7)  Burkur et al., 2017 (8)  Brooks & Peetz (9) | Netherlands, USA |
| Acute Care Geriatric Unit | Boyer et al., 1986 (10)  Bellizzi 2018 (11)  Palmer 2018 (12) | USA |
| Surgical post-acute treatment unit (SPE) | Digiacomo et al., 2019 (13) | USA |
| Orthogeriatric care | Neurenberg et al., 2019 (14) | Germany |
| Orthogeriatric co-management (OGC) | Baroni et al., 2018 (15) | Italy |
| Geriatric fracture center | Blauth et al., 2021 (16)  Mendelson & Friedman, 2014 (17) | USA, Austria, Switzerland, Netherlands, Spain, Germany, Thailand, Singapore |
| Geriatric trauma Center | Halvachizadeh et al., 2021 (18) | Switzerland |
| Orthogeriatric service | Wyller et al., 2012 (19)  Watne et al., 2014 (20) | Norway |
| Orthogeriatric Trauma Unit | Schuijt et al., 2020 (21)  Folbert et al., 2017 (22) | Netherlands |
| Integrated hip fracture service | Middleton 2018 (23) | UK |
| Orthogeriatric Unit | Kristensen et al., 2016 (24) Gonzalez et al., 2011 (25) | Denmark, Spain |
| Sheba model | Adunsky et al., 2002 (26), 2005 (27) | Israel |
|  |  |  |

**References**

1. DeLa'O CM, Kashuk J, Rodriguez A, Zipf J, Dumire RD. The Geriatric Trauma Institute: reducing the increasing burden of senior trauma care. The American Journal of Surgery. 2014;208(6):988-94.
2. Kates S, Mendelson D, Friedman S. Co-managed care for fragility hip fractures (Rochester model). Osteoporosis international. 2010;21:621-5.
3. Lorenzo M, Barba C. Will patient outcomes improve if we admit and manage geriatric patients in a specialized unit in the trauma center. Abstract presentation Jackson Hole, Wyoming: Western Trauma Association. 2005.
4. Lorenzo M, Shifflette V. Geriatric Trauma Service: What is so Special? Current Surgery Reports. 2020;8:1-8.
5. Leede E, Fry L, Crosby L, Hamilton S, Ali S, Brown C. Impact of geriatric trauma service on the outcome of older trauma patents. Geriatrics & Gerontology International. 2020;20(9):817-21.
6. Mangram AJ, Mitchell CD, Shifflette VK, Lorenzo M, Truitt MS, Goel A, et al. Geriatric trauma service: a one-year experience. Journal of Trauma and Acute Care Surgery. 2012;72(1):119-22.
7. Mangram AJ, Shifflette VK, Mitchell CD, Johnson Jr VA, Lorenzo M, Truitt MS, et al. The creation of a geriatric trauma unit “G-60”. The American Surgeon. 2011;77(9):1144-6.
8. Bukur M, Catino J, Puente I, Farrington-Avila R, Crawford M, Habib F. The G-60 trauma center: a future consideration? Journal of the American College of Surgeons. 2014;219(4):e54.
9. Brooks SE, Peetz AB. Evidence-based care of geriatric trauma patients. Surgical Clinics. 2017;97(5):1157-74.
10. Boyer N, Chuang J, Gipner D. An acute care geriatric unit. Nursing Management. 1986;17(5):22-5.
11. Bellizzi M. An ACE for South Philly: Acute Care for the Elderly (ACE) Unit Makes All the Difference at Methodist Hospital. Population Health Matters (Formerly Health Policy Newsletter). 2018;31(2):12.
12. Palmer RM. The acute care for elders unit model of care. Geriatrics. 2018;3(3):59.
13. DiGiacomo JC, Angus LG, Cardozo-Stolberg S, Wallace R, Gerber N, Munnangi S, et al. Betwixt and between: a surgical post-acute treatment unit (SPA) for the optimal care of elderly patients with isolated hip fractures. Aging Clinical and Experimental Research. 2019;31:1743-53.
14. Neuerburg C, Förch S, Gleich J, Böcker W, Gosch M, Kammerlander C, et al. Improved outcome in hip fracture patients in the aging population following co-managed care compared to conventional surgical treatment: a retrospective, dual-center cohort study. BMC geriatrics. 2019;19(1):1-11.
15. Baroni M, Serra R, Boccardi V, Ercolani S, Zengarini E, Casucci P, et al. The orthogeriatric comanagement improves clinical outcomes of hip fracture in older adults. Osteoporosis International. 2019;30:907-16.
16. Blauth M, Joeris A, Rometsch E, Espinoza-Rebmann K, Wattanapanom P, Jarayabhand R, et al. Geriatric fracture centre vs usual care after proximal femur fracture in older patients: what are the benefits? Results of a large international prospective multicentre study. BMJ open. 2021;11(5):e039960.
17. Mendelson DA, Friedman SM. Principles of comanagement and the geriatric fracture center. Clinics in geriatric medicine. 2014;30(2):183-9.
18. Halvachizadeh S, Gröbli L, Berk T, Jensen KO, Hierholzer C, Bischoff-Ferrari HA, et al. The effect of geriatric comanagement (GC) in geriatric trauma patients treated in a level 1 trauma setting: A comparison of data before and after the implementation of a certified geriatric trauma center. PLoS One. 2021;16(1):e0244554.
19. Wyller TB, Watne LO, Torbergsen A, Engedal K, Frihagen F, Juliebø V, et al. The effect of a pre-and post-operative orthogeriatric service on cognitive function in patients with hip fracture. The protocol of the Oslo Orthogeriatrics Trial. BMC geriatrics. 2012;12(1):1-13.
20. Watne LO, Torbergsen AC, Conroy S, Engedal K, Frihagen F, Hjorthaug GA, et al. The effect of a pre-and postoperative orthogeriatric service on cognitive function in patients with hip fracture: randomized controlled trial (Oslo Orthogeriatric Trial). BMC medicine. 2014;12:1-12.
21. Schuijt HJ, Kusen J, van Hernen JJ, van der Vet P, Geraghty O, Smeeing DPJ, et al. Orthogeriatric trauma unit improves patient outcomes in geriatric hip fracture patients. Geriatric Orthopaedic Surgery & Rehabilitation. 2020;11:2151459320949476.
22. Folbert E, Hegeman J, Gierveld R, Van Netten J, Velde Dvd, Ten Duis H, et al. Complications during hospitalization and risk factors in elderly patients with hip fracture following integrated orthogeriatric treatment. Archives of orthopaedic and trauma surgery. 2017;137:507-15.
23. Middleton M. Orthogeriatrics and hip fracture care in the UK: factors driving change to more integrated models of care. Geriatrics. 2018;3(3):55.
24. Kristensen PK, Thillemann TM, Søballe K, Johnsen SP. Can improved quality of care explain the success of orthogeriatric units? A population-based cohort study. Age and ageing. 2016;45(1):66-71.
25. González-Montalvo JI, Alarcón T, Mauleón JL, Gil-Garay E, Gotor P, Martín-Vega A. The orthogeriatric unit for acute patients: a new model of care that improves efficiency in the management of patients with hip fracture. Hip International. 2010;20(2):229-35.
26. Adunsky A, Levi R, Cecic A, Arad M, Noy S, Barell V. The" Sheba" model of comprehensive orthogeriatric care for elderly hip fracture patients: a preliminary report. IMAJ-RAMAT GAN-. 2002;4(4):259-61.
27. Adunsky A, Arad M, Levi R, Blankstein A, Zeilig G, Mizrachi E. Five-year experience with the ‘Sheba’model of comprehensive orthogeriatric care for elderly hip fracture patients. Disability and rehabilitation. 2005;27(18-19):1123-7.
