# Supplementary material for: Exploring geriatric trauma unit experiences through patients’ eyes: a qualitative study
Source: BMC Geriatr. 2024 May 30;24:476. doi: 10.1186/s12877-024-05023-z (PMC11140891; doi:10.1186/s12877-024-05023-z)
Supplement: Supplementary file 3 — Supplementary Material 3 [file 12877_2024_5023_MOESM3_ESM.docx]

# Interview guide

| **General** | - Can you tell me how you came to fall?   **Prompts**:  How are you experiencing the care in the Geriatric Trauma Unit? |
| --- | --- |
| **TOPICS** | |
| **Information, communication and Education** | \| Could you share your initial expectations about the care provided?  Which information was discussed with you regarding this care?  Among the information you received, what did you find most significant or impactful?  **Follow-up prompt Prompts:**  By whom was the information provided, and how clear was their communication?  Did the HPs take the tome to explain things to you (and your family)?  Overall, how satisfied are you with the information provided? \| \| --- \| |
| **Respect for patient values, preferences, and needs** | Can you describe your interactions with the nurses, doctors, and other healthcare professionals?  In what ways would you like to be more involved in your own care planning and decision-making?  Could you highlight some of the most positive aspects of the care you've received?  **Follow-up prompts:**  Was the impact of the treatment on your everyday life considered? Could you explain how this was addressed?"  Who was involved in your care, and how was each person's role explained to you?  How were your capabilities and limitations taken into account during your treatment?  What are your specific preferences and needs in terms of your care, and how were these addressed by the healthcare team?  How were your personal treatment goals established and followed up on? |
| **Physical comfort** | Over the past few days, how have you been feeling (in terms of pain, your ability to conduct activities of daily living (ADLs), and your nutrition)?  Could you share your recent experiences with performing ADL?  What is your opinion on the facilities available at the GTU? |
| **Emotional support** | What did HPs do to comfort you?  Can you describe the impact that the fall had on your overall well-being  **Follow-up prompts:**  Were any feelings of anxiety, sadness, or fear of falling addressed by the healthcare team? How was this handled? |
| **Involvement of family and friends** | How were decisions made?  How was the family involved in care? Did you find this helpful?  **Follow-up prompts:**  How did HPs acknowledge the care and support provided by family  Was there adequate attention to the questions and needs of family members |
| **Coordination and integration of care** | Do you feel that HPs are well-informed about your condition?  How is your care coordinated among different HPs?  Whom can you approach for inquiries?  **Prompts:**  Which HPs have you encountered? |
| **Continuity and transitional care?** | Can you discuss the next steps in the rehabilitation process?  **Prompts:**  How was this discussed with you?  How were the recommendations from various practitioners coordinated (e.g. nurse, geriatrician, surgeon, physiotherapist etc.)  If input from other disciplines was needed, were they readily available? |
| **Closure** | - Is there anything else you would like to add to the conversation? - Are there any important points that have not been addressed but you would like to mentions? |
| **Member check** | - What is your feedback on the interview? - Would you like to receive a copy of the transcribed interview? |
